# PCMMD: A Novel Dataset of Plasma Cells to Support the Diagnosis of Multiple Myeloma

**DOI:** 10.1038/s41597-025-04459-1

**Published:** 2025-01-27

**Authors:** Caio L. B. Andrade, Marcos V. Ferreira, Brenno M. Alencar, Jorge L. S. B. Filho, Matheus A. Guimaraes, Iarley Porto Cruz Moraes, Tiago J. S. Lopes, Allan S. dos Santos, Mariane M. dos Santos, Maria I. C. S. e Silva, Izabela M. D. R. P. Rosa, Gilson C. de Carvalho, Herbert H. M. Santos, Márcia M. L. Santos, Roberto Meyer, Luciana M. P. B. Knop, Songeli M. Freire, Ricardo A. Rios, Tatiane N. Rios

**Affiliations:** 1 Federal University of Bahia, Institute of Computing, Salvador, 40170-110 Brazil; 2 Federal University of Bahia, Institute of Health Sciences, Salvador, 40231-300 Brazil; 3 Nezu Biotech GmbH, Heidelberg, 69121 Germany; 4 Federal University of Bahia, Hospital Universitario Professor Edgard Santos - HUPES, Salvador, 40110-060 Brazil; 5 Hospital Martagão Gesteira, LABCMI-HMG, Salvador, 40050-050 Brazil

**Keywords:** Oncogenesis, Myeloma

## Abstract

Multiple Myeloma (MM) is a cytogenetically heterogeneous clonal plasma cell proliferative disease whose diagnosis is supported by analyses on histological slides of bone marrow aspirate. In summary, experts use a labor-intensive methodology to compute the ratio between plasma cells and non-plasma cells. Therefore, the key aspect of the methodology is identifying these cells, which relies on the experts’ attention and experience. In this work, we present a valuable dataset comprising more than 5,000 plasma and non-plasma cells, labeled by experts, along with some patient diagnostics. We also share a Deep Neural Network model, as a benchmark, trained to identify and count plasma and non-plasma cells automatically. The contributions of this work are two-fold: (i) the labeled cells can be used to train new practitioners and support continuing medical education; and (ii) the design of new methods to identify such cells, improving the results presented by our benchmark. We emphasize that our work supports the diagnosis of MM in practical scenarios and paves new ways to advance the state-of-the-art.

## Background & Summary

Multiple Myeloma (MM) is a hematological malignancy that originates from a clone of malignant plasma cells (mPCs) infiltrated in the bone marrow (BM)^1,2^. The MM diagnosis is confirmed by the presence of ≥10% of clonal plasma cells (PCs) in the bone marrow (or plasmacytoma confirmed by biopsy), and by the occurrence of end-organ damage such as hypercalcemia (serum calcium  > 0.25 mmol/L,  > 1 mg/dL, higher than the upper limit of normal or  > 2.75 mmol/L,  > 11 mg/dL), renal insufficiency (creatinine clearance  < 40 mL per min† or serum creatinine  > 177 *μ*mol/L,  > 2 mg/dL), anemia (haemoglobin value of  > 20 g/L below the lower limit of normal, or a haemoglobin value  < 100 g/L), and bone lesions (one or more osteolytic lesions on skeletal radiography, CT, or PET-CT)^3^.

Regarding the assessment of the 10% ratio between plasma and non-plasma cells, experts individually analyze hematological slides to identify and count these cells^4^, encountering several challenges even when using advanced equipment to compute them, such as: 1) manual identification of plasma cells on histological slides requires significant time and resources, potentially delaying diagnosis and treatment; 2) the identification process strongly depends on the expert’s attention and experience, leading to inconsistencies in result interpretation, thereby compromising the sensitivity and overall diagnostic accuracy; and 3) in resource-constrained communities, there is often a lack of access to trained professionals, significantly barring effective disease screening and diagnostic practices.

Therefore, considering the challenges associated with manually identifying plasma cells, we have employed a low-cost setup consisting of a microscope and a smartphone camera to create a comprehensive dataset of over 5,000 plasma and non-plasma cells manually labeled by experts.

Our dataset, PCMMD (Plasma Cells for Multiple Myeloma Diagnosis)^5^, is an important resource for training and validating Artificial Intelligence (AI) approaches to automate cell identification, supporting studies on MM in developing countries, and discovering approaches regarding other diseases affecting various cell types in diverse population conditions. In previous work^6^, plasma cells were labeled as part of a preliminary study. However, this dataset had several limitations, including a small number of cells, lack of information on other non-plasma cells, absence of patient diagnoses, and insufficient details about the cells’ nuclei, shapes, and membrane structures. In this manuscript, we address all these limitations. Moreover, the computational findings presented here serve as a benchmark, demonstrating the feasibility and effectiveness of AI-assisted diagnosis in hematological studies, with distinct contributions to the field:

- The labeled-cell dataset assists with training new practitioners and continuing medical education, ensuring that future experts can accurately identify plasma cells;
- Using a Deep Neural Network (DNN) model, our Machine Learning approach provides a benchmark that encourages the development of new AI methods and technologies that surpass the current state-of-the-art methods in cell identification;
- Combining microscopes and smartphone cameras, our AI model offers a low-cost, accessible solution for diagnosing MM, making advanced diagnostic techniques available in resource-constrained settings;
- The availability of our dataset and benchmark model support ongoing research and development in the field, promoting continuous improvement in the accuracy and efficiency of MM diagnostics.

In summary, our work improves the practical diagnosis of MM and opens new avenues for advancing the field through improved training, innovative method development, and enhanced accessibility.

## Methods

The dataset was created in accordance with the relevant ethical regulations established by the Institutional Research Committee - CEP ICS (CAAE - 70193723.5.0000.5662) from the Federal University of Bahia. The research ethics committee have waived the necessity for researchers to obtain informed consent from patients.

The methodology considered in this work adapted the CRISP-DM (CRoss-Industry Standard Process for Data Mining) process, which is a standard widely adopted in Machine Learning projects^7^. In the first step, we defined the problem by focusing on using images from histological slides to support experts in diagnosing MM, as discussed in the previous section. The second step comprises the tasks performed to collect, preprocess, and analyze such images. In the third step, we trained a DNN model to automatically identify and count plasma and non-plasma cells. After validating the results, in the fourth step, we deployed the model to assess its capability of suggesting diagnoses to patients with known positive and negative conditions, according to experts. In the following section, we provide more details about these steps.

### Collection and Preparation

This section details the second step of CRISP-DM, which focuses on collecting images extracted from bone marrow (BM) histological slides and organizing the MM dataset. In this study, a team of hematology experts, including two hematologists, two biomedical hematologists and immunologists, and one biotechnologist immunologist, selected patients with confirmed oncohematological diseases, including MM.

All these patients were diagnosed and treated by the Brazilian Public Health System (Sistema Único de Saúde - SUS)^8–11^. The diagnosis by immunophenotyping was carried out in the Oncohematology and Immunophenotyping sector of the Laboratory of Immunology and Molecular Biology at the Federal University of Bahia (UFBA), which provided the samples for the present study. We emphasize that the slides were coded, respecting the users’ privacy, and categorized into groups of patients with and without MM.

**Fig. 1:**
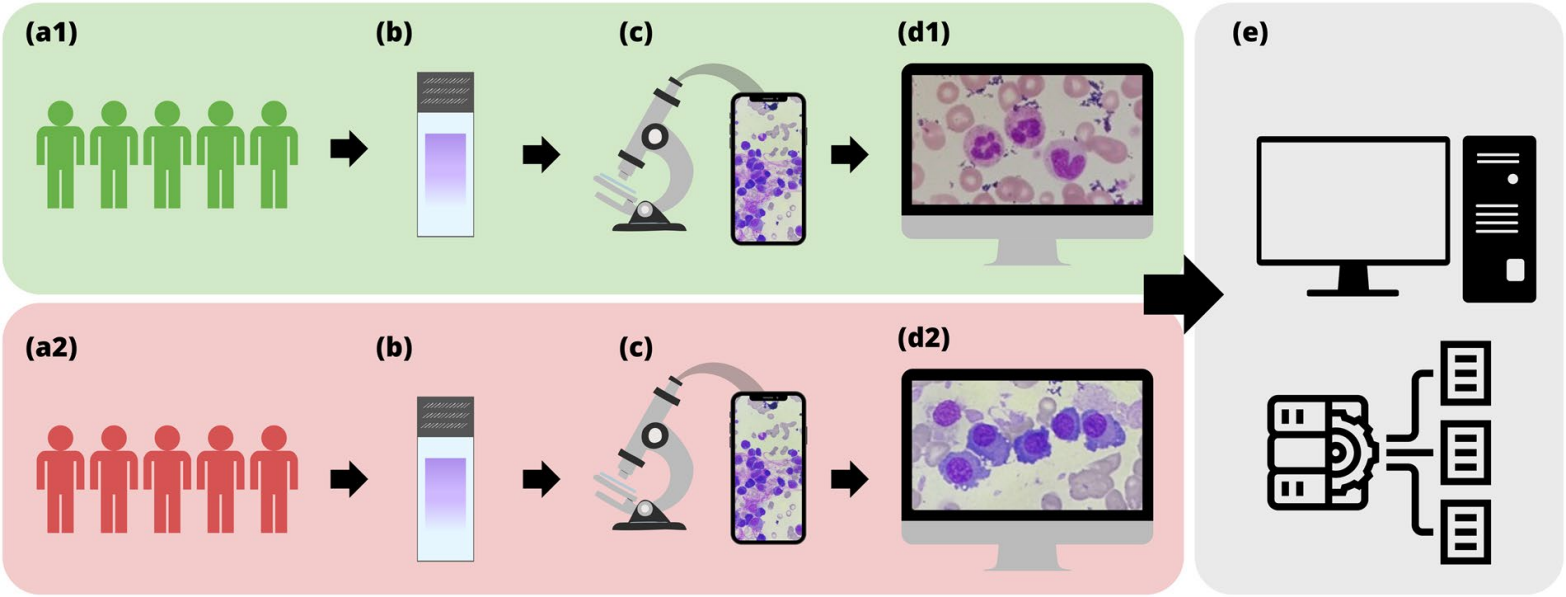
(**a1**) Bone marrow aspirate procedure of patients without MM; (**a2**) Bone marrow aspirate procedure of patients with MM; (**b**) Wright-Giemsa (SIGMA-ALDRICH, MERCK) stained bone marrow aspirate smear slides, analyzed by the oncohematology and immunophenotyping service of the Labimuno; (**c**) Observation of stained slides in visible light optical microscopes and image capture by smartphone device; (**d1** and **d2**) Identification, labeling and validation of detected non-plasma and plasma cells from patients with and without MM; and (**e**) Dataset organization to be later trained by DNN models.

In Fig. 1, we illustrate the entire collection and preparation process. In the first stage, groups of patients with (Stage a2) and without (Stage a1) MM, who were submitted to a bone marrow aspirate procedure, thus resulting in a set of histological slides (Stage b). The BM slides were examined using a Nikon ECLIPSE CI visible light optical microscope with immersion oil, employing a 100x objective lens and a 10x ocular lens (Stage c). Observations were conducted at the smear’s feathered edge. Nucleated cells were photographed using a smartphone camera mounted on the microscope with a universal holder. Notably, our dataset comprises both mono- and multinucleated cells. In the last stage, cell images from patients with (Stage d2) and without MM (Stage d1) were individually analyzed and labeled, as depicted in the next section, to be later modeled by our DNN architectures.

### Cell Labeling

After analyzing and validating the collected images, our expert team was divided into two groups to label all cells as plasma and non-plasma. This process was carried out using the LabelImg tool^12^. Each group manually labeled a separate set of images and validated the labels set by the other group.

**Fig. 2:**
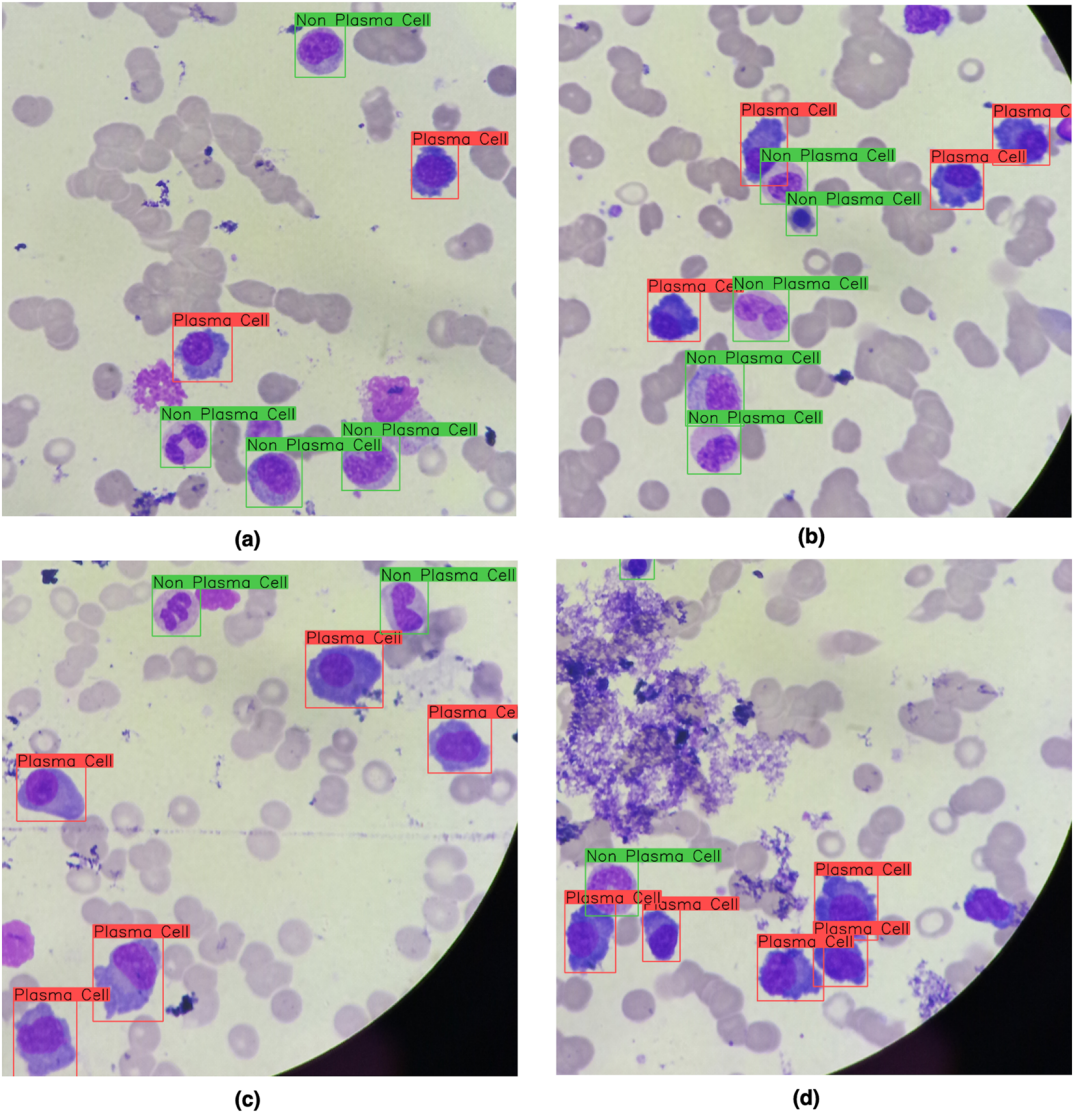
Examples of stained cells annotated by experts, with plasma cells marked by red bounding boxes and non-plasma cells marked by green bounding boxes.

The labeling process was performed based on cytomorphological characteristics and Wright-Giemsa staining to determine each identifiable cell type. Figure 2 shows 4 examples of images with labeled cells, highlighting plasma (in red) and non-plasma (in green) bounding boxes.

In Fig. 2(a), we show 4 non-plasma and 2 plasma cells labeled by our experts. Moreover, one may also notice a set of red cells (not labeled) as background. In Figure 2(b) and (c), it is possible to realize two artifacts not labeled for being partially cut by the top-most and left-most image limits, respectively. In this situation, experts tend to disregard such artifacts to avoid misclassification. In Fig. 2(d), there are regions, also considered artifacts, affected by the staining process. In the next section, we present more details about the final dataset.

### Dataset Description

The dataset is organized into two sets of cells. The first one contains 3,546 cells, of which 54% were labeled as non-plasma and 46% as plasma cells. This set was used to train our DNN model, as detailed in the following section. Each cell was individually segmented to remove background elements, enabling the analysis of key features such as the number of nuclei, cell shapes, and membrane structures. From this segmentation process, 1,613 plasma cells and 1,927 non-plasma cells were identified and shared as complementary data.

The second set contains 2,021 cells that we have used to test the performance of our model. Besides measuring the agreement between labels provided by experts and predicted by our model, we also separated the cells according to the patients’ diagnoses, as shown in Table 1. According to the literature, the MM diagnosis depends on counting 200-500 cells in myelogram slides of bone marrow (BM) aspirate. Columns Non-Plasma Cells, Plasma Cells, and Total Cells show the number of cells in each patient’s slide. If more than 10% are plasma cells (Percentage column), it indicates a positive diagnosis. In our investigation, we have considered at least 200 cells to create the dataset with patients diagnosed with (diseased) and without (healthy) MM, in which hematologist experts carefully curated all images. In the following section, we describe the experimental setup considered in this work.

**Table 1:** Diagnostic dataset.

Patient	Non-Plasma Cells	Plasma Cells	Total Cells	Percentage	Target
Patient 01	120	82	202	40.6%	diseased
Patient 02	127	76	203	37.4%	diseased
Patient 03	128	72	200	36.0%	diseased
Patient 04	116	82	198	41.4%	diseased
Patient 05	129	71	200	35.5%	diseased
Patient 06	185	15	200	7.5%	healthy
Patient 07	187	18	205	8.8%	healthy
Patient 08	199	9	208	4.3%	healthy
Patient 09	188	14	202	6.9%	healthy
Patient 10	188	15	203	7.4%	healthy

This table provides an overview of cell distribution among ten patients, detailing the counts of non-plasma cells, plasma cells, and total cells. It also includes the percentage of plasma cells in relation to total cells and the corresponding the patients’ diagnoses (diseased or healthy).

### Experimental Setup

We have designed two experimental setups to assess the relevance of our dataset. In the first one, we have used the labeled cells, as illustrated in Fig. 2, to train a DNN model capable of learning patterns from such cells. In our second experimental setup, we analyzed the possibility of automatically suggesting diagnoses to patients (Table 1) based on the ratio of plasma cells in myelogram slides.

Our model allows automatic analysis of images from slides of BM aspirate to detect and identify plasma and non-plasma cells, thus supporting hematologists during the diagnosis procedure. Details about our DNN model are discussed in the following section.

The evaluation methodology was based on conventional practices used in computer vision (CV) for object detection tasks. Our study assessed the bounding boxes surrounding cells defined by the experts (ground truth) and those predicted by our model. By considering the bounding boxes, it is possible to compute the Intersection Over Union (IOU) ratio as defined in Equation (1) to estimate the number of true positives (TP), false positives (FP), and false negatives (FN). This estimation is based on a threshold, which works as an interval for accepting or rejecting a predicted bounding box. In brief, if *IOU* ≥ *τ*_1_, there is enough overlapping between the predicted and ground truth bounding boxes, thus classifying the detected cell as true positive (TP). If the predicted bounding box does not have enough overlapped area with ground truth, a false positive (FP) is detected. Similarly, when a prediction bounding box does not overlap a ground truth area, it is considered a false negative (FN).

1 $$IOU=\frac{G\cap P}{G\cup P}$$

**Fig. 3:**
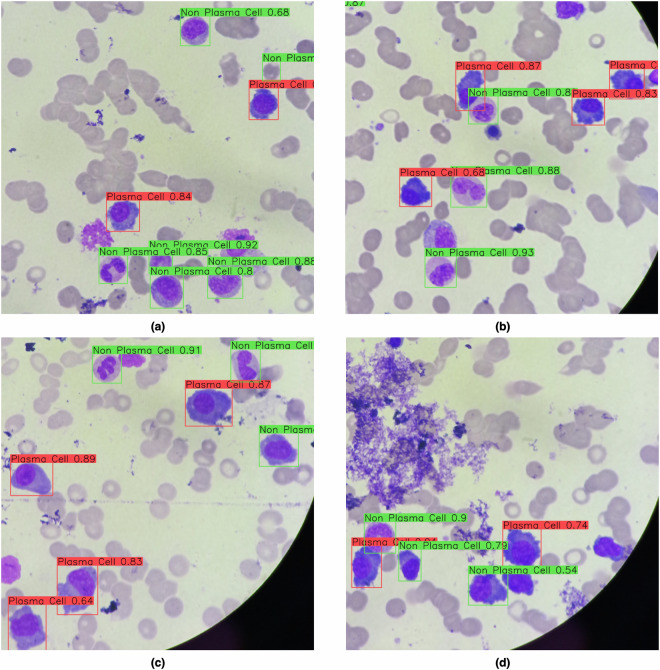
Example of stained cells labeled by our DNN model as plasma (red) and non-plasma (blue). The label probabilities were also plotted along with the bounding box captions. The ground truth bounding boxes for these cells were shown in Fig. 2.

In Fig. 3, we show 4 images to visually illustrate the performance of our DNN, in which the bounding boxes contain the predicted labels and their respective probability. The ground truth for all cells presented in these images was previously shown in Fig. 2. Based on these figures, it is possible to exemplify different classification performances in terms of TP, FP, and FN estimated from IOU.

For example, in Fig. 3(a), we can notice a TP prediction considering the top-most detected non-plasma cell. There is an overlapping between the prediction and expected (Fig. 2(a)) bounding boxes. These figures also show an example of FP prediction, by analyzing the cluster of three non-plasma cells in Fig. 2(d). In contrast, the DNN model, Fig. 3(a), predicted four non-plasma cells on the same region. In the center of Fig. 2(b), we can notice a cluster with one plasma and two non-plasma cells. In our prediction, Fig. 3(b), we noticed two examples of TP, but one non-plasma cell (the smaller one) is missing, exemplifying a FN. In Fig. 3(c), we illustrate a situation in which all expected cells were correctly detected in Fig. 2(c). However, a plasma cell was misclassified as non-plasma cell by our model (Fig. 3(c)). Finally, in Fig. 3(d), we illustrate a FN prediction, once our DNN was not capable of detecting a plasma cell in the right-most cluster presented in Fig. 2(d).

Based on IOU, we also assess the model confidence (*C*) by measuring the performance of predicting a cell in a bounding box. In summary, this confidence is calculated by the product between the probability f existing a cell and IoU, such as *C* = Pr(Object) * IOU. Considering the confidence, the model accepts the predicted bounding box when *C* ≥ *τ*_2_. The thresholds *τ*_1_ and *τ*_2_ are hyperparameters (not trainable), which can be varied to find objects in images better. Once there is no well-defined approach to set their values, we used a grid search in our experiments and selected their best combination based on the performance measured with F1-score, which is detailed next.

After calculating IOU, we compute three metrics widely used in CV tasks to assess the performance of our model: Precision, Recall, and F1-score. In summary, Precision (Equation (2)) computes the rate of correct classifications for the positive label over the number of outcomes classified as positives. On the other hand, Recall (Equation (3)) measures the models’ completeness, which is calculated by the rate of correct classifications for the positive label over the number of elements expected to be under the positive label. F1-score works as a harmonic mean of precision and recall, as shown in Equation (4).

2 $${\rm{Precision}}=\frac{TP}{TP+FP}$$

3 $${\rm{Recall}}=\frac{TP}{TP+FN}$$

4 $$\,{\rm{F1-score}}\,=\frac{2\times {\rm{Precision}}\times {\rm{Recall}}}{{\rm{Precision}}+{\rm{Recall}}}$$

### Deep learning architecture

To automatically detect plasma cells in our new dataset, we have used a DNN architecture based on YOLO (You Only Look Once), a popular real-time object detection technique. YOLO consists of three main components: the backbone, the neck, and the head. The backbone extracts features from images at varying levels of detail. These features are then passed through the neck, which improves processing efficiency by combining different scales and reducing dimensionality before reaching the head component for final object detection.

We have used the version Ultralytics YOLOv8^13^, which is well-suited for real-time object detection tasks across various application areas by maintaining an optimal balance between accuracy and speed. This version incorporates state-of-the-art backbone and neck architectures, enhancing feature extraction and object detection performance. YOLOv8 also utilizes an anchor-free split Ultralytics head, which improves accuracy and efficiency compared to anchor-based methods. The head component detects objects, draws bounding boxes, and infers the estimated classes (labels), by using a self-attention mechanism, enabling the model to focus on various parts of the image and adjust the importance of different features based on their relevance to the task.

Over time, the backbone component of the YOLO series has evolved through several architectures: Darknet in the first version^14^, Cross-Stage Partial Network (CSP) in version 5^15^, Extended Efficient Layer Aggregation Network (E-ELAN) in version 7^16^, and CSPDarknet53 in version 8^13^. CSPDarknet53 uses a split and merge strategy to improve the flow of gradients through the network, helping to improve the system’s accuracy by allowing it to learn more complex representations of the input data.

Another significant contribution of YOLOv8 is the model scaling, which allows the network to be adjusted on different devices and applications. Considering this functionality, we configured and personalized a DNN to distinguish between plasma and non-plasma cells in stained bone marrow aspirate smear slides. This configuration process involved the traditional construction of a DNN, including adding and removing layers, adjusting image resolution, modifying channels and filters, and tuning various parameters.

We also implemented transfer learning by initializing our models with pre-trained weights and then training them on our custom dataset. These weights are derived from a model trained on the COCO (Common Objects in Context)^17^ dataset, a large-scale dataset commonly used for object detection, segmentation, and captioning tasks in computer vision. By reusing the knowledge learned from previous tasks, we enhanced the performance of detecting plasma cells. Finally, we conducted a fine-tuning process on our images, which significantly improved our results in detecting bounding box sizes, probabilities, and classifications of plasma cells.

The final configuration of our training process was obtained after using a batch with a size equal to 32, 300 epochs, 12 GB of GPU memory, a learning rate equal to 0.01, and an Adam optimizer. After using our images during the training phase, the resultant model presented 225 layers with 3,157,200 parameters.

## Data Records

The dataset is available at Mendeley Data^5^. The data folder is organized into two subfolders: “detection” and “segmentation”. The detection one contains randomized stained slide images and patient-specific datasets, which include the slides utilized for diagnostic analysis. In addition to the labeled images, this folder also provides the configuration files used for training the DNN models, following a cross-validation strategy.

**Fig. 4:**
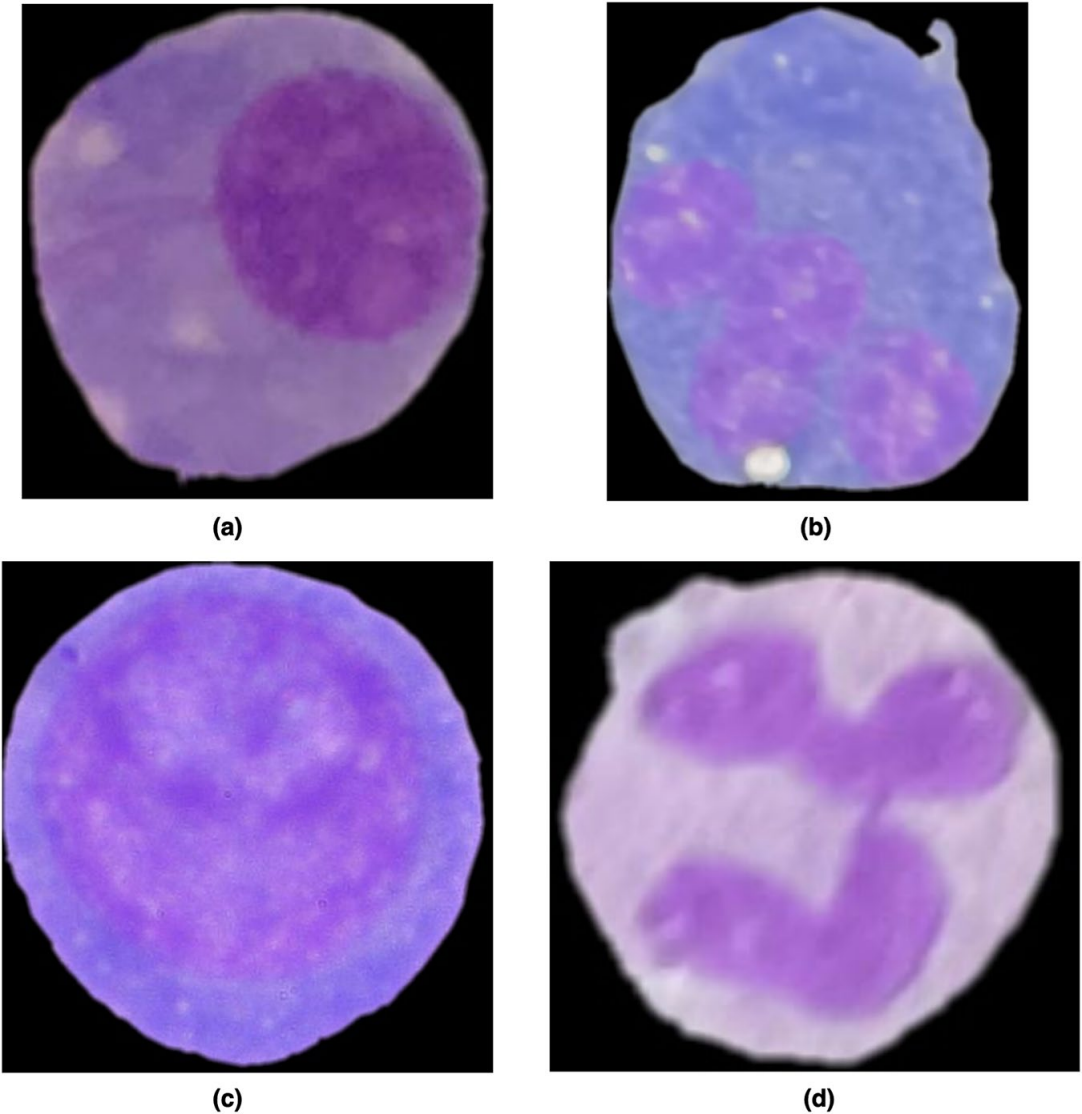
Examples of segmented cells: (**a**) a plasma cell with a single nucleus; (**b**) a plasma cell containing multiple nuclei; (**c**) a non-plasma cell with a single nucleus; and (**d**) a non-plasma cell with segmented nucleus.

The primary objective of this work is to differentiate plasma and non-plasma cells without using additional cell features such as the number of nuclei, shapes, and membrane structures. A key direction for future work involves developing models capable of learning from these features, as they hold the potential to provide deeper insights into the disease dynamics and uncover critical properties related to MM. To achieve this objective, the proposed dataset includes an subfold called “segmentation” containing numerous plasma and non-plasma cells segmented from the original stained slides, as illustrated in Fig. 4.

Beyond being utilized to train models capable of learning cellular features, this dataset also enables the development of generative AI models that can create synthetic cells resembling real ones. These synthetic cells can enhance the quality of DNNs, provide a richer variety of examples for model training, and support the education of new practitioners. This segmentation process was carried out using the AnyLabeling (https://anylabeling.nrl.ai/). Our dataset consists of the following files: image data in JPG format, label information in TXT format, segmentation coordinates in JSON format, cross-validation configurations in TXT and YAML files, and diagnosis data in CSV format.

## Technical Validation

**Fig. 5:**
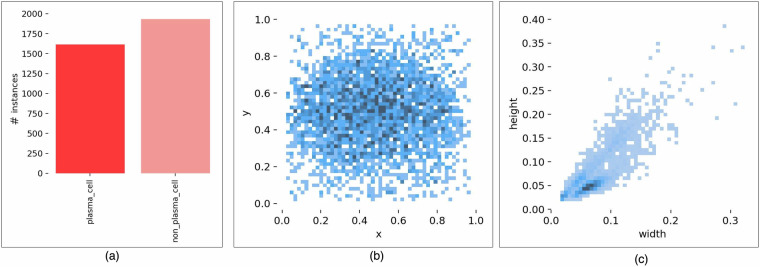
Label and bounding box distributions over slide images: (**a**) the number of cells per label; (**b**) the distribution of cells across the slide images; and (**c**) the shape (width  × height) of the bounding boxes.

After creating the dataset, we conducted a thorough validation process from two perspectives. First, we performed a visual analysis of the cell distribution across all images from the stained slides, as illustrated in Fig. 5. Figure 5(a) displays the number of cells per label, while Fig. 5(b) summarizes the distribution of cells across the slide images. As expected, the slides are positioned on the microscope to centralize the clusters of cells. These figures are crucial for visualizing the overall placement of plasma and non-plasma cells in our dataset. Lastly, Fig. 5(c) illustrates the shape (width  × height) of the bounding boxes, emphasizing the regions surrounding the labeled cells.

From the second perspective, we trained a DNN model to demonstrate the feasibility of extracting patterns and learning from our images. This process highlights the technical quality of our dataset and illustrates the potential for using both stained slides and segmented cells to enhance diagnostic processes and develop new AI-based methods to support specialists.

Initially, we focused our attention on the first experimental setup, which aims to detect and identify plasma and non-plasma cells. Using the DNN architecture previously defined, we started the training process using the 5-fold cross-validation strategy to find the optimal configuration for our DNN and to avoid obtaining results by chance. In Table 2, we present the cell distributions by labels and folds. In each fold, different cell configurations are used to train (train) the model and validate (val) its performance. As one may notice, we also used a nearly balanced amount of data to avoid inducing better results for a specific class.

**Table 2:** The table presents the distribution of non-plasma and plasma cells across the training (train) and validation (val) sets for each of the five folds used in the model training process.

Fold	Non-Plasma Cells		Plasma Cells	
	train	val	train	val
1	1626	305	1298	317
2	1448	483	1290	325
3	1461	470	1291	324
4	1627	304	1289	326
5	1562	369	1292	323

Each fold ensures a balanced split between training and validation data, contributing to robust cross-validation.

Considering the train and validation cells, the obtained mean F1-score for all five folds was 78%. This result suggests our model was learning from the training data. The individual results obtained for each training and validation fold are available in the supplementary material.

**Fig. 6:**
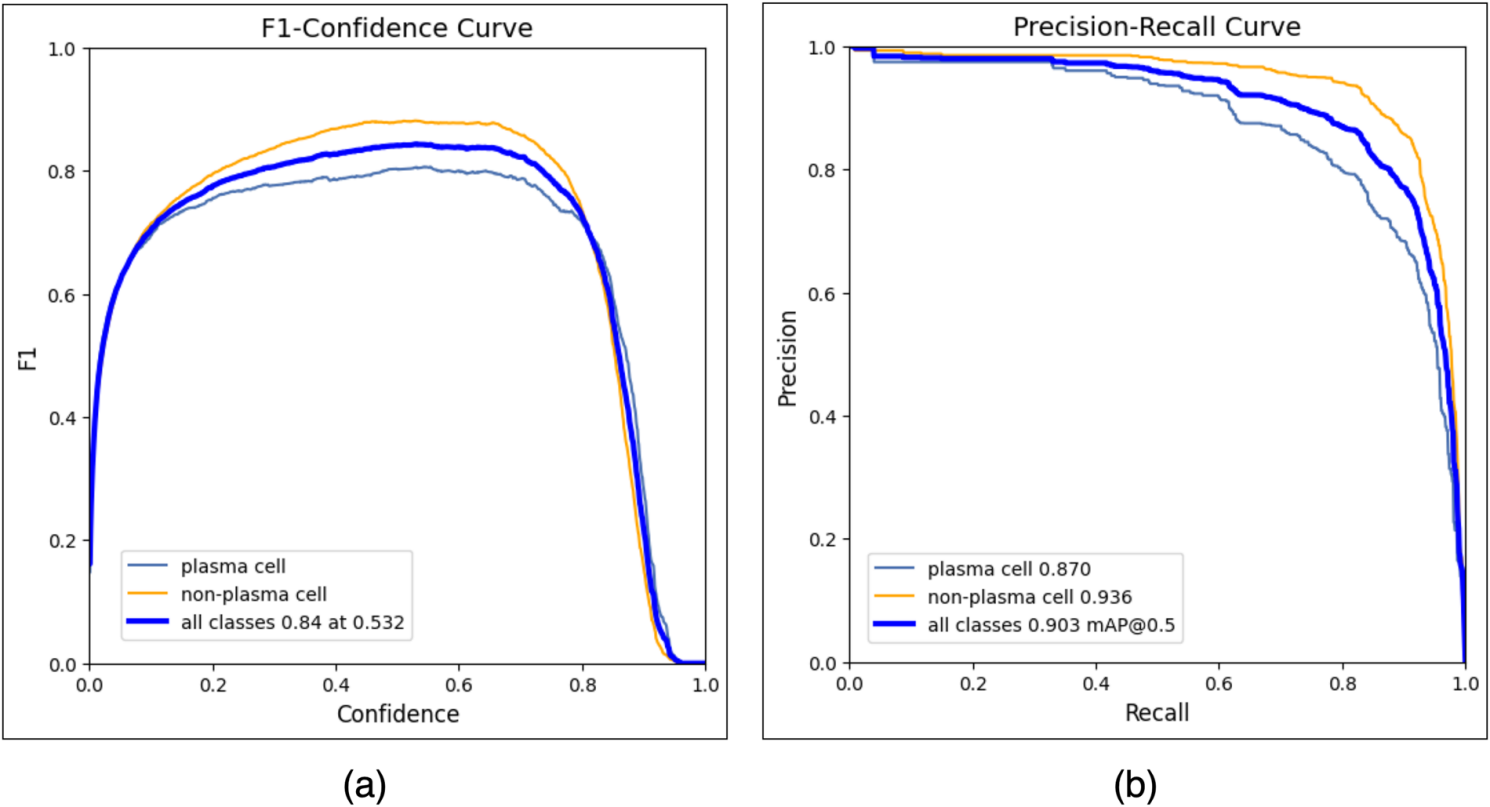
(**a**) Best F1-score obtained by varying the confidence threshold. The best performance (84%) was obtained using a threshold equal to 0.532. (**b**) Precision and recall curve to illustrate the learning process. The best performance (93.5%) was obtained using mAP50.

In the next step, we used the entire dataset to find the optimal configuration to classify cells in our second experimental setup, i.e., analyzing the Diagnostic dataset presented in Table 1. In this sense, we have executed a grid search to better combine the thresholds and confidences. In our experiment, we noticed similar results were produced by varying *τ*_1_; hence, we set it as *τ*_1_ = 0.7. However, the relationship between *τ*_2_ and *C* significantly affects the final classification, as illustrated in Fig. 6(a). In our experiments, the best performance to detect and identify plasma and non-plasma cells achieved F1-score = 84% with *τ*_2_ = 0.532.

**Fig. 7:**
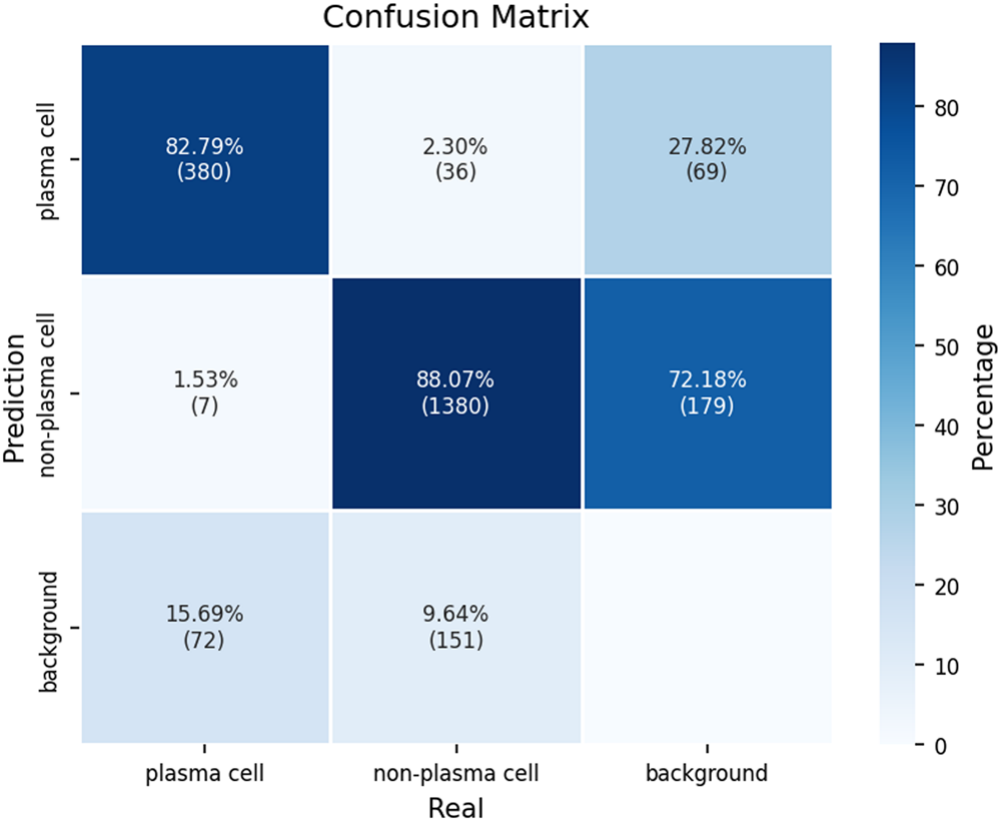
Confusion (contingency) matrix showing the classification performance of the DNN model to predict the three classes: plasma_cell, non_plasma_cell, and background. Correct classifications are represented along the diagonal, while misclassifications are shown in the off-diagonal elements. The color gradient indicates the percentage of predictions, with darker shades corresponding to higher performances.

This F1 score indicates that our DNN model demonstrates significant overall performance in classifying cells. Next, we assessed its performance on each individual class. In Fig. 7, we present the relation between expected (true or ground truth) and predicted bounding boxes as a confusion (a.k.a. contingency) matrix.

The diagonal results for plasma and non-plasma cells emphasize that most bounding boxes were correctly predicted (true positives, TP). False positives (FP) and false negatives (FN) are represented by the other values in this matrix. According to this matrix, 381 plasma cells were correctly identified by our DNN model, with only 37 non-plasma cells misclassified as plasma cells. Regarding non-plasma cells, 1,378 were correctly classified, while only 7 were misclassified as plasma cells.

In relation to the background, 71 areas were incorrectly marked as plasma cells and 152 as non-plasma cells. These numbers are considerably low, as the protocol followed by specialists avoids drawing bounding boxes around cells that are not fully visible. Nevertheless, even when partially omitted or not fully visible, our DNN model can still identify cells. Although this may increase classification errors, it does not pose a significant issue, as such cases are typically disregarded during the diagnostic process.

In Fig. 6(b), we show the relevant performance of our DNN model, presenting a mAP50 result superior to 90%. Considering all analyses summarized in these figures, we are confident that our dataset contains valuable patterns to identify plasma and non-plasma cells, providing an important and low-cost setup to support hematologists. Moreover, our dataset can also be used to train new hematologists, providing different cell patterns.

The final challenge presented in this work lies in verifying the performance of our approach in effectively aiding specialists in diagnosing MM. To reach this goal, we analyzed slides for each patient in Table 3. In summary, we have used our DNN model to detect, identify, and count plasma and non-plasma cells. Next, we calculate the ratio between them. As aforementioned, if the ratio is greater than 10%, the MM diagnosis is positive. As one may notice, there is narrow agreement between the expert and predicted diagnoses. The only different was found in Patient 07, in which the ratio was 8.8% and the ratio predicted by our model was 11.1%. However, once were are using the DNN model to support experts, we consider it a less serious mistake compared to incorrectly labeling a patient’s exam with MM as healthy (negative for MM). In this case, the expert is warned to recount the cells.

**Table 3:** Comparison of model-predicted disease status with expert diagnosis for 10 patients.

Patient	Non-Plasma Cells	Plasma Cells	Total Cells	Percentage	Expert Diagnosis	Model Diagnosis
Patient 01	119	77	196	39.3%	diseased	diseased
Patient 02	121	83	204	40.7%	diseased	diseased
Patient 03	124	77	201	38.3%	diseased	diseased
Patient 04	99	71	170	41.8%	diseased	diseased
Patient 05	108	70	178	39.3%	diseased	diseased
Patient 06	197	18	215	8.4%	healthy	healthy
Patient 07	208	26	234	11.1%	healthy	diseased
Patient 08	238	15	253	5.9%	healthy	healthy
Patient 09	209	15	224	6.7%	healthy	healthy
Patient 10	197	14	211	6.6%	healthy	healthy

The table also presents the counts of non-plasma and plasma cells, total cell count, and the percentage of plasma cells.

To better understand the performance of the DNN model in every patient, Table 4 shows the precision, recall, and F1 scores. These metrics allow for an individual assessment of the model’s effectiveness, highlighting its strong performance on cell slides collected from both healthy and diseased patients.

**Table 4:** Performance metrics for the learning model evaluated per patient.

Patient	Precision	Recall	F1 Score
Patient 01	0.863	0.848	0.855
Patient 02	0.868	0.865	0.866
Patient 03	0.854	0.837	0.845
Patient 04	0.885	0.746	0.810
Patient 05	0.866	0.773	0.817
Patient 06	0.726	0.768	0.746
Patient 07	0.786	0.770	0.778
Patient 08	0.756	0.878	0.812
Patient 09	0.910	0.906	0.908
Patient 10	0.920	0.800	0.856

These metrics allow for an individual assessment of the model’s effectiveness, highlighting its strong performance on cell slides collected from both healthy and diseased patients.

Our contribution is distinct from usual approaches due to focusing on modeling images captured using standard smartphone cameras. This feature is particularly pertinent as it facilitates the decision-making process in hospitals with resource-constrained settings. Moreover, we also provide a fine-tuned DNN model capable of detecting and identifying plasma and non-plasma cells. This model is especially important to support hematologists and scientists devoted to using it as a benchmark to improve the state-of-the-art in DNN to detect cells. Further results using the DNN architecture are provided in the supplementary material.

## Usage Notes

The dataset is under license Creative Commons (CC) BY 4.0. We encourage all interested researchers to download and utilize this dataset for detecting and identifying plasma cells, as well as investigating new machine learning methods for classifying patients with MM.

## Supplementary information

PCMMD: A Novel Dataset of Plasma Cells to Support1 the Diagnosis of Multiple Myeloma (Supplementary File)

## Data Availability

The source code, models, and datasets used in the study are freely available at https://github.com/LabIA-UFBA/MMDB. The repository is structured into two primary folders: “src”, which contains all source code and the DNN model utilized in this study, and “data”, which includes all cell data shared in this research. Besides this dataset, we also created a repository with all codes used to preprocess the cells, organize the experiments, and train our DNN model. Such codes are available at https://github.com/LabIA-UFBA/MMDB. Aiming to assure the reproducibility of our results, the reader can find in our repositories all configurations to split the dataset into train, validate, and test folds. Notebooks with codes illustrating every step of our investigation are also available to easily support researchers interested in deeply understanding our work. Finally, in supplementary material, we present a datasheet that provides general information on our dataset.
