# Supplementary material for: PCMMD: A Novel Dataset of Plasma Cells to Support the Diagnosis of Multiple Myeloma
Source: Sci Data. 2025 Jan 27;12:161. doi: 10.1038/s41597-025-04459-1 (PMC11772599; doi:10.1038/s41597-025-04459-1)
Supplement: Supplementary file 1 — PCMMD: A Novel Dataset of Plasma Cells to Support1 the Diagnosis of Multiple Myeloma (Supplementary File) [file 41597_2025_4459_MOESM1_ESM.pdf]

PCMMD: A Novel Dataset of Plasma Cells to Support the Diagnosis of Multiple Myeloma

Caio L. B. Andrade[1,2], Marcos V. Ferreira[1], Brenno M. Alencar[1], Jorge L. S. B. Filho[1], Matheus A. Guimaraes[1], Iarley Porto Cruz Moraes[1], Tiago J.S. Lopes[3], Allan S. dos Santos[2], Mariane M. dos Santos[2], Maria I. C. S. e Silva[2], Izabela M. D. R. P. Rosa[2], Gilson C. de Carvalho[2], Herbert H. M. Santos[2], Márcia M. L. Santos[4], Roberto Meyer[2], Luciana M. P. B. Knop[5], Songeli M. Freire[2], Ricardo A. Rios[1,*], and Tatiane N. Rios[1]

[1]Federal University of Bahia, Institute of Computing, Salvador, 40170-110, Brazil
[2]Federal University of Bahia, Institute of Health Sciences, Salvador, 40231-300, Brazil
[3]Nezu Life Sciences, Karlsruhe, 76149, Germany
[4]Federal University of Bahia, Hospital Universitario Professor Edgard Santos - HUPES, Salvador, 40110-060, Brazil
[5]Hospital Martagão Gesteira, LABCMI-HMG, Salvador, 40050-050, Brail.
[*]ricardoar@ufba.br

ABSTRACT

Supplementary information with dataset details and results obtained with our benchmark using a Deep Neural Network.

Contents

1 Dataset General Information **1**
1.1 Dataset and Benchmark Access . . . 1
1.2 Data Sheet . . . 1
Motivation • Distribution • Maintenance • Composition • Collection Process • Uses
2 Learning Models **4**
2.1 Fold 1 . . . 6
Label and bounding box distributions • F1-score Results • Confusion Matrix Results • Training Performance • Precision-Recall Curve
2.2 Fold 2 . . . 11
Label and bounding box distributions • F1-score Results • Confusion Matrix Results • Training Performance • Precision-Recall Curve
2.3 Fold 3 . . . 16
Label and bounding box distributions • F1-score Results • Confusion Matrix Results • Training Performance • Precision-Recall Curve
2.4 Fold 4 . . . 21
Label and bounding box distributions • F1-score Results • Confusion Matrix Results • Training Performance • Precision-Recall Curve
2.5 Fold 5 . . . 26
Label and bounding box distributions • F1-score Results • Confusion Matrix Results • Training Performance • Precision-Recall Curve

1 Dataset General Information

1.1 Dataset and Benchmark Access

To support the maintenance of our contributions, we have shared all data and code in a GitHub repository (`https://github.com/LabIA-UFBA/MMDB`). The data includes all raw and graph-based datasets. The code consists of scripts used to preprocess and transform the raw datasets, as well as models used as benchmarks. We emphasize that the code contains notebooks with commands organized into a structure that supports better comprehension of our work and the reproducibility of our results.

1.2 Data Sheet

1.2.1 Motivation

1. **For what purpose was the dataset created?** *Our dataset is an important resource for training and validating Artificial Intelligence (AI) approaches to automate cell identification, supporting studies on multiple myeloma (MM) in developing*

countries, and discovering approaches regarding other diseases affecting various cell types in diverse population conditions.

2. **Who created the dataset and on behalf of which entity?** *The dataset was created by researchers from different fields at the the Federal University of Bahia in Brazil in collaboration with researchers from Hospital Martagão Gesteira in Brazil and Nezu Life Sciences in Germany.*

3. **Who funded the creation of the dataset?** *This work was supported by Terumo Life Science Foundation grant 406354/2023-5; CNPq (Brazilian National Council for Scientific and Technological Development) grants [312755/2023-6, 313053/2023-5]; Maria Emilia Foundation grant to 01/2023; INCITE FAPESB grant TO PIE0002/2022; CAPES (Coordination for the Improvement of Higher Education Personnel—Brazilian federal government agency) finance code 001; FAPESB (Bahia Research Foundation) grant [1589/2021]; Google Research Awards for Latin America 2021; Council for Science, Technology and Innovation (CSTI); Cross-ministerial Strategic Innovation Promotion Program (SIP); 'Innovative AI Hospital System'; National Institute of Biomedical Innovation, Health and Nutrition (NIBIOHN) [grant number SIPAIH20D01]; JSPS KAKENHI [JP22K06119]; and National Center for Child Health and Development internal grant [2022B-2].*

1.2.2 Distribution

1. **Will the dataset be distributed to third parties outside of the entity (e.g., company, institution, organization) on behalf of which the dataset was created?** *Yes, the dataset is entirely open to the public.*

2. **How will the dataset be distributed (e.g., tarball on website, API, GitHub)?** The dataset, codes, and models are already available at https://github.com/LabIA-UFBA/MMDB.

3. **Have any third parties imposed IP-based or other restrictions on the data associated with the instances?** *No.*

4. **Do any export controls or other regulatory restrictions apply to the dataset or to individual instances?** *No.*

1.2.3 Maintenance

1. **Who will be supporting/hosting/maintaining the dataset?** *The GitHub will host the dataset. The authors will support and maintain the dataset as new data is collected, labelled and validated over time.*

2. **How can the owner/curator/manager of the dataset be contacted (e.g., email address)?** *The owner/curator/manager(s) of the dataset can be contacted through the following email of the corresponding author (ricardoar@ufba.br).*

3. **Is there an erratum?***No. If any errors are discovered in the future, we will publish errata on the main page of our GitHub repository.*

4. **Will the dataset be updated (e.g., to correct labeling errors, add new instances, delete instances)?** *Yes, we will update the dataset as necessary to maintain its correctness and up-to-date information. Announcements will be made accordingly. We will publish the dataset versions on the main web page of the GitHub repository.*

5. **If the dataset relates to people, are there applicable limits on the retention of the data associated with the instances (e.g., were the individuals in question told that their data would be retained for a fixed period of time and then deleted)?** *The dataset does not contain personal information. Project approved by the Institutional Research Committee – CEP ICS (CAAE - 70193723.5.0000.5662). The request for waiver of signing the Termo de Consentimento Livre Esclarecido - TCLE (Informed Consent Form) to the Conselho de Ética em Pesquisa - CEP (Research Ethics Council) was justified by the use of slides and data tabulated and coded by specialists in the diagnosis of MM in the clinical care hematological and laboratorial sectors of health care at a public university. This request was to encourage the procedure, since the evidence comes from sets of bone marrow (BM) aspirate slides from the bank of coded slides and integrated into the collections of MM diagnoses under the custody of hematologists and technicians from the diagnostic service at Hospital and Laboratory. This request by the Investigators avoids substantial risks to privacy and breach of trust between the physician, patient and investigator-researcher.*

6. **Will the older version of the dataset continue to be supported/hosted/maintained?***Yes, all dataset versions will be continuously maintained and hosted.*

1.2.4 Composition

1. **What do the instances that comprise the dataset represent (e.g., documents, photos, people, countries)?** *The dataset contains photos of microscopical images of slides of bone marrow aspirate, of onco-hematological disease patients.*
2. **How many instances are there in total (of each type, if appropriate)?** *The final dataset is organized into two sets of cells. The first one contains 3,546 cells, of which 54% were labeled as non-plasma and 46% as plasma cells. This set was used to train our DNN model. The second set contains 2,021 cells, of which 78% were labeled as non-plasma and 22% as plasma cells, that we have used to test the performance of our model.*
3. **Does the dataset contain all possible instances or is it a sample of instances from a larger set?** *The dataset contains all instances considering the collected up to June 2024.*
4. **Is there a label or target associated with each instance?** *There are two labels which may occur in each instance. The labels available are "plasma cell" and "non-plasma cell".*
5. **Is any information missing from individual instances?** *No.*
6. **Are there recommended data splits (e.g., training, development/validation, testing)?** *No.*
7. **Are there any errors, sources of noise, or redundancies in the dataset?** *No.*
8. **Is the dataset self-contained, or does it link to or otherwise rely on external resources (e.g., websites, tweets, other datasets)?** *The dataset is self-contained.*
9. **Does the dataset contain data that might be considered confidential?** *No.*
10. **Does the dataset contain data that, if viewed directly, might be offensive, insulting, threatening, or might otherwise cause anxiety?** *No.*

1.2.5 Collection Process

1. **What mechanisms or procedures were used to collect the data (e.g., hardware apparatus or sensor, manual human curation, software program, software API)?** *In the first stage, groups of patients with and without MM, who were submitted to a BM aspirate procedure, thus resulting in a set of histological slides. The BM slides were visualized using a Nikon ECLIPSE CI visible light optical microscope in immersion oil with an optical zoom of 100x objective and 10x ocular lens. The observation was performed at the smear's feathered edge. The nucleated cells were photographed using a smartphone camera mounted on the microscope with a universal holder. In the last stage, cell images from patients with and without MM were individually analyzed, labeled and validated by a team of experts, using the software Labelimg.*
2. **Who was involved in the data collection process (e.g., students, crowdworkers, contractors), and how were they compensated (e.g., how much were crowdworkers paid)?** *For the data collection process, a team of hematology experts from the Federal University of Bahia, including two hematologists and two biomedical hematologists and immunologists, who work in the public health service of onco-hematological diagnoses, and one biotechnologist immunologist researcher, assisted by two biotechnology graduate students.*
3. **Does the dataset relate to people?** *Yes.*
4. **Did you collect the data from the individuals in question directly or obtain it via third parties or other sources (e.g., websites)?** *The data was provided by the Oncohematology and Immunophenotyping sector of the Laboratory of Immunology and Molecular Biology (Labimuno) at the Federal University of Bahia (UFBA), of patients who were diagnosed and treated by the National Health Service (Sistema Único de Saúde - SUS). We emphasize that the slides were coded, respecting the user's privacy, and categorized into groups of patients with and without MM.*

1.2.6 Uses

1. **Has the dataset been used for any tasks already?** *The dataset has been used only for the learning tasks related to BM cells identification and MM diagnoses by myelogram.*
2. **What (other) tasks could the dataset be used for?** *Further use of the dataset includes graph-based optimization problems, multi-objective optimization, and learning tasks using Concept Drift, among other possibilities. Please refer to Section* **??** *for detailed future works.*
3. **Is there anything about the composition of the dataset or the way it was collected and preprocessed/cleaned/labeled that might impact future uses?** *No, there is not.*
4. **Are there tasks for which the dataset should not be used?** *No.*

2 Learning Models

This section provides details about the results obtained in every step of our 5-fold cross-validation. More information about the experimental setup, result discussion, and validation approaches is available in the manuscript.

To understand better our model's performance during training, we present a set of plots in Figure 1 that illustrate the learning progress over different epochs. The first row displays training metrics, while the second row illustrates validation metrics.

In addition to precision and recall, which were discussed previously, we also include traditional metrics used for training Artificial Neural Networks (ANNs). Typically, the training process is evaluated using loss and accuracy curves. Generally, the loss curve decreases as the number of epochs increases, whereas the accuracy tends to increase. A brief introduction to loss is provided in our supplementary material. In CV projects, it is common to compute the area under the precision-recall curve (AP) instead of using accuracy. Extending this concept, mAP calculates the average AP values. In our experiments, we adopted mAP50 and mAP50-95, such that mAP50 uses an IoU threshold of 0.50, and mAP50-95 uses a range of IoU thresholds from 0.50 to 0.95. Similarly to accuracy, mAP50 and mAP50-95 tend to increase as new training epochs is considered.

The box-loss (train/box_loss and val/box_loss) curves represent the error in predicted bounding boxes compared to the ground truth annotations provided by specialists. Both training and validation box-loss curves demonstrate a steep decline during the initial epochs, followed by stabilization, indicating the model's effective learning of object localization. The classification-loss (train/cls_loss and val/cls_loss) curves summarize the categorical errors in object classification. Initially, the loss is high but progressively decreases for both training and validation, reflecting consistent improvements in the model's classification accuracy. The distance-field-loss (train/dfl_loss and val/dfl_loss) curves capture fine-grained details of bounding box predictions. Like the other loss curves, its steady reduction suggests successful optimization of localization precision, further supporting the model's effective learning process.

In a different perspective, the precision, recall, and mAP curves tends to increase as the results are improved. The precision (metrics/precision(B)) curve for the bounding box predictions steadily increases, achieving high values as new ephocs are calculated, showing low false-positive rates in the model predictions. Similarly, the recall (metrics/recall(B)) curve improves over time, reflecting the model's capability of detecting the majority of the objects with minimal false negatives. Finally, the mean Average Precision (metrics/mAP50(B) and metrics/mAP50-95(B)) curves allow to analyze the overall detection performance at varying the IoU thresholds. Both curves show a consistent upward trend, confirming progressive learning and generalization with minimal overfitting.

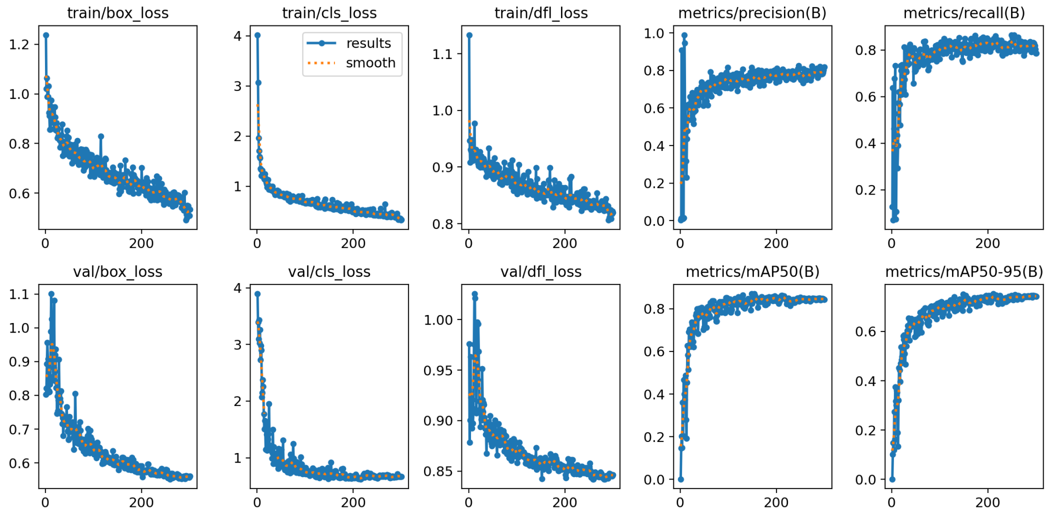

Figure 1. Training and validation curves for box loss (box_loss), classification loss (cls_loss), and distance field loss (dfl_loss) alongside precision, recall, and mAP metrics. The results indicate consistent learning and generalization, with losses decreasing and metrics improving over epochs.

Besides providing the results for each fold, it is important to briefly present an introduction to loss functions. The loss functions used to train ANN-based models depends on the analyzed task. For binary classification tasks, we have considered Binary Cross-Entropy (BCE) as defined in Equation (1), assuming the possible labels are $\{0, 1\}$. In this equation, X^T is a set of instances selected to test the model, $y_i \in X^T$ is the class for the i-th tested instance, and $\hat{y}_i$ is its respective prediction.

$$\mathscr{L}_{\text{BCE}} = -\frac{1}{|X^T|}\sum_{i=1}^{|X^T|} y_i \log(\hat{y}_i) + (1-y_i)\log(1-\hat{y}_i) \tag{1}$$

In case of classification tasks with more classes, we have used Cross-Entropy (CE) defined in Equation (2), such that C represents all possible classes.

$$\mathscr{L}_{\text{CE}} = -\frac{1}{|X^T|}\sum_{i=1}^{|X^T|}\sum_{c=1}^{|C|} y_{i,c} \log(\hat{y}_{i,c}) \tag{2}$$

2.1 Fold 1

2.1.1 Label and bounding box distributions

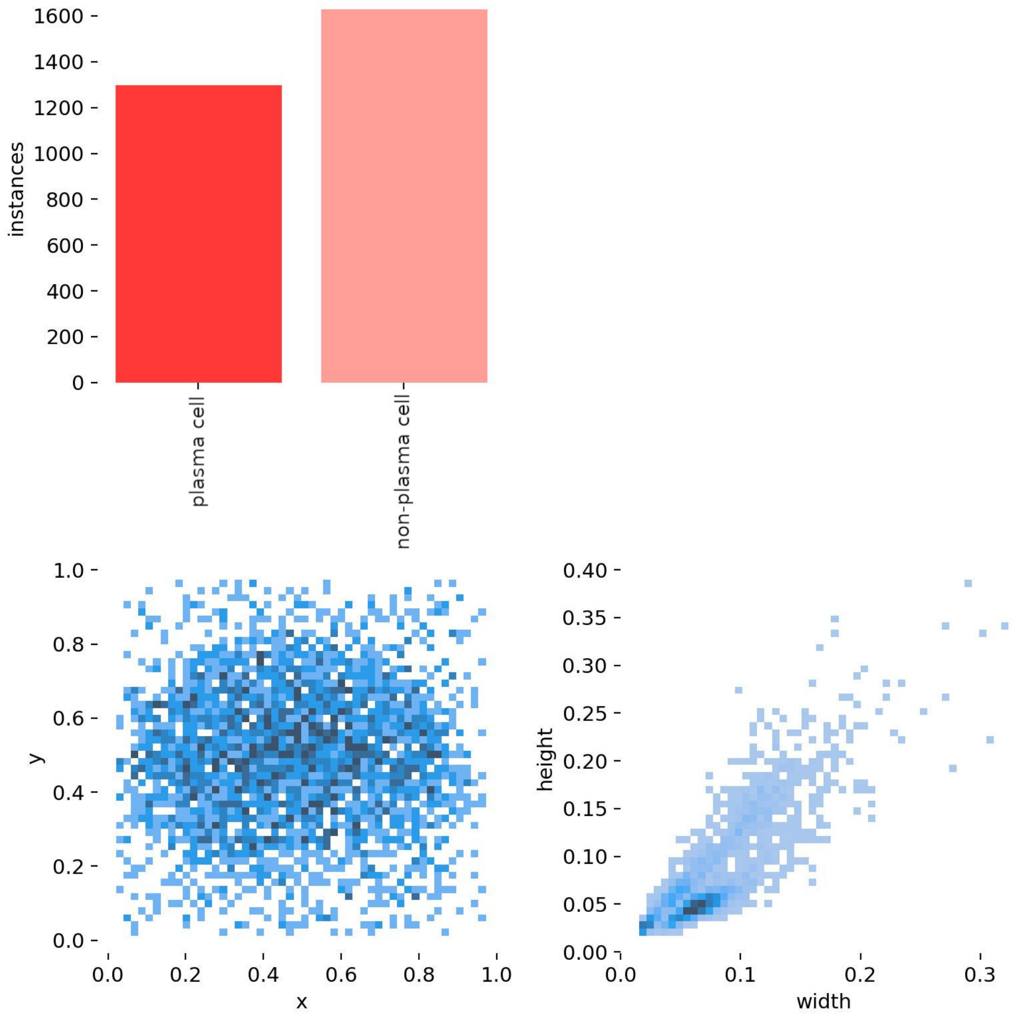

Figure 2. Label and bounding box distributions over slide images.

2.1.2 F1-score Results

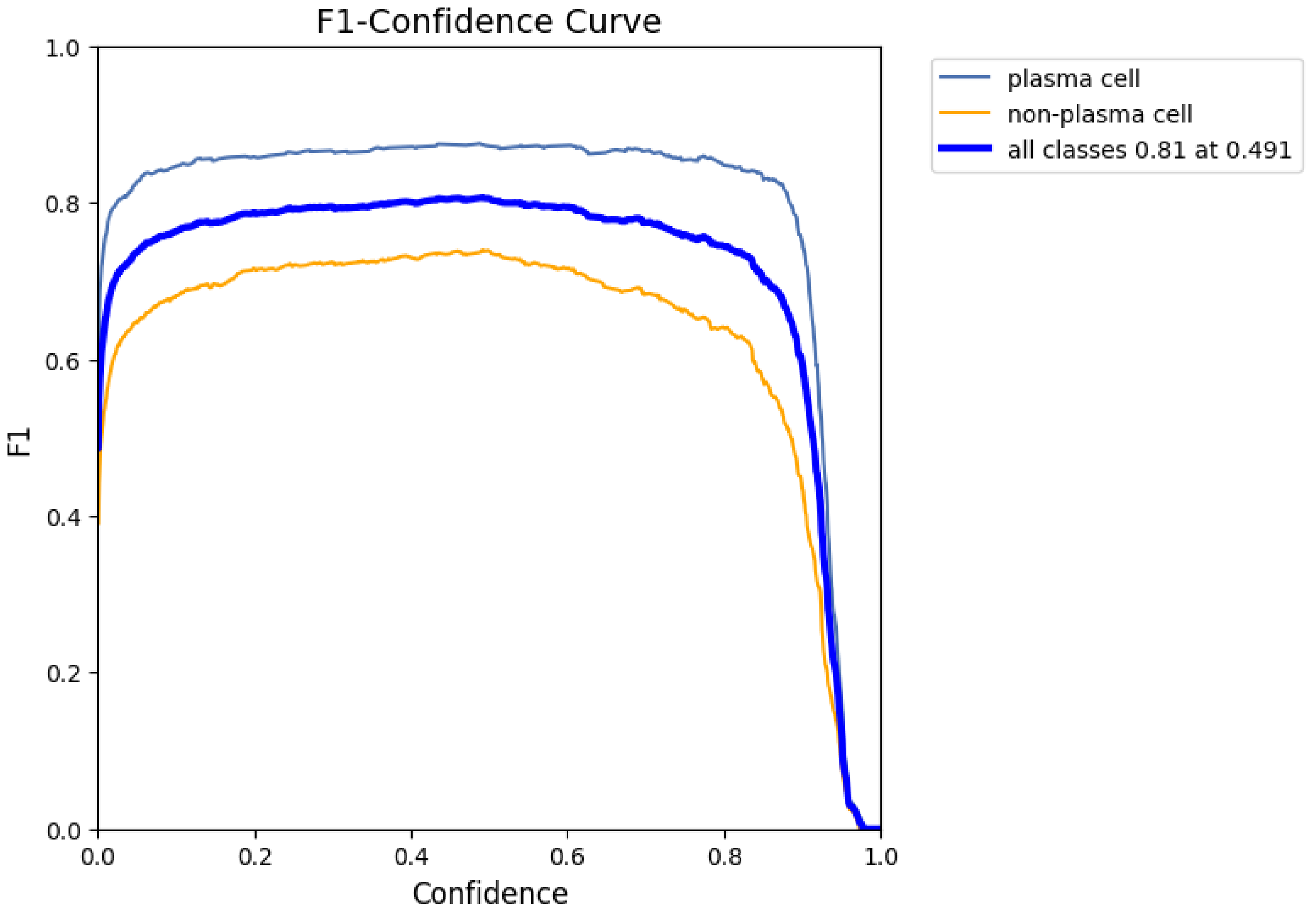

Figure 3. Best F1-score obtained by varying the confidence threshold.

2.1.3 Confusion Matrix Results

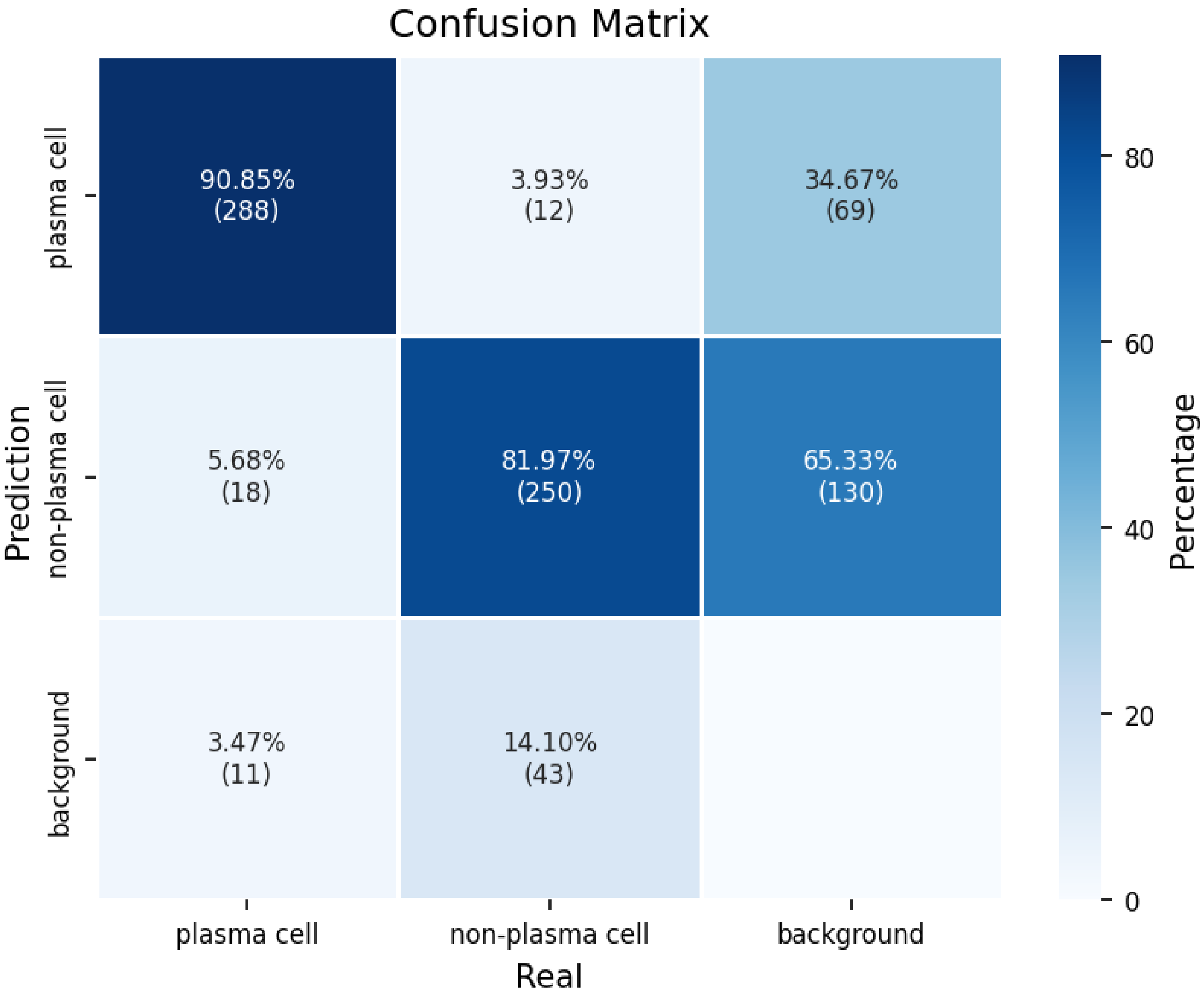

Figure 4. Confusion matrix summarizing the performance of our DNN model in each class.

2.1.4 Training Performance

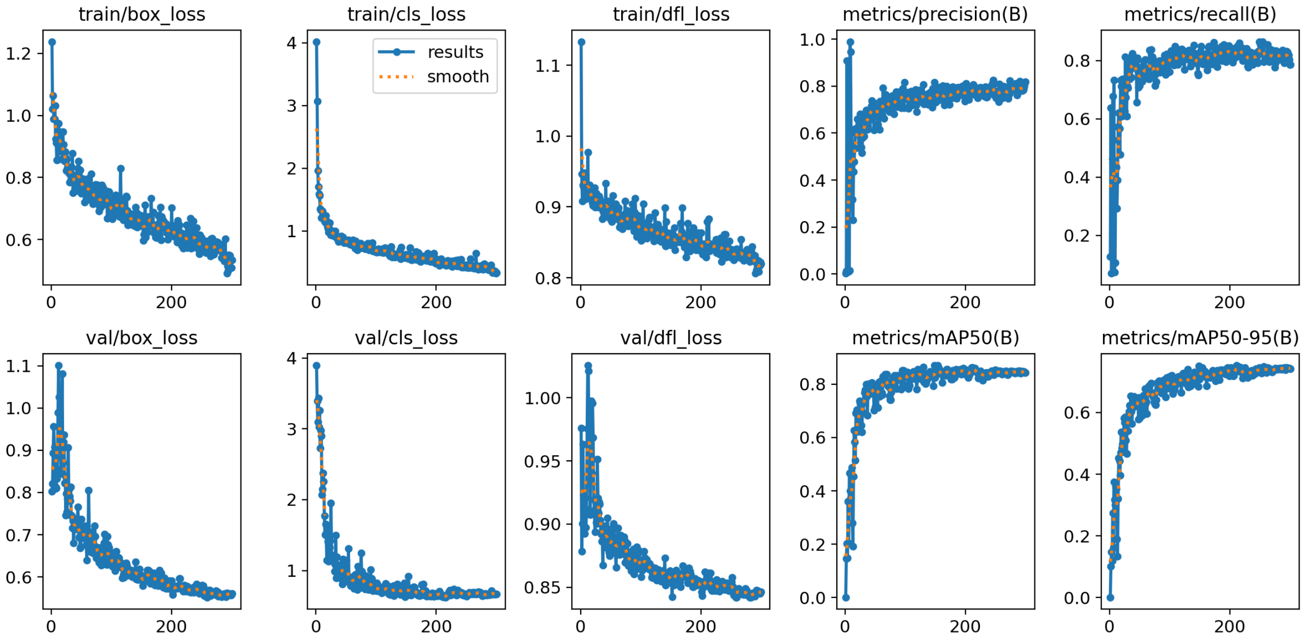

Figure 5. General training and validation performances.

2.1.5 *Precision-Recall Curve*

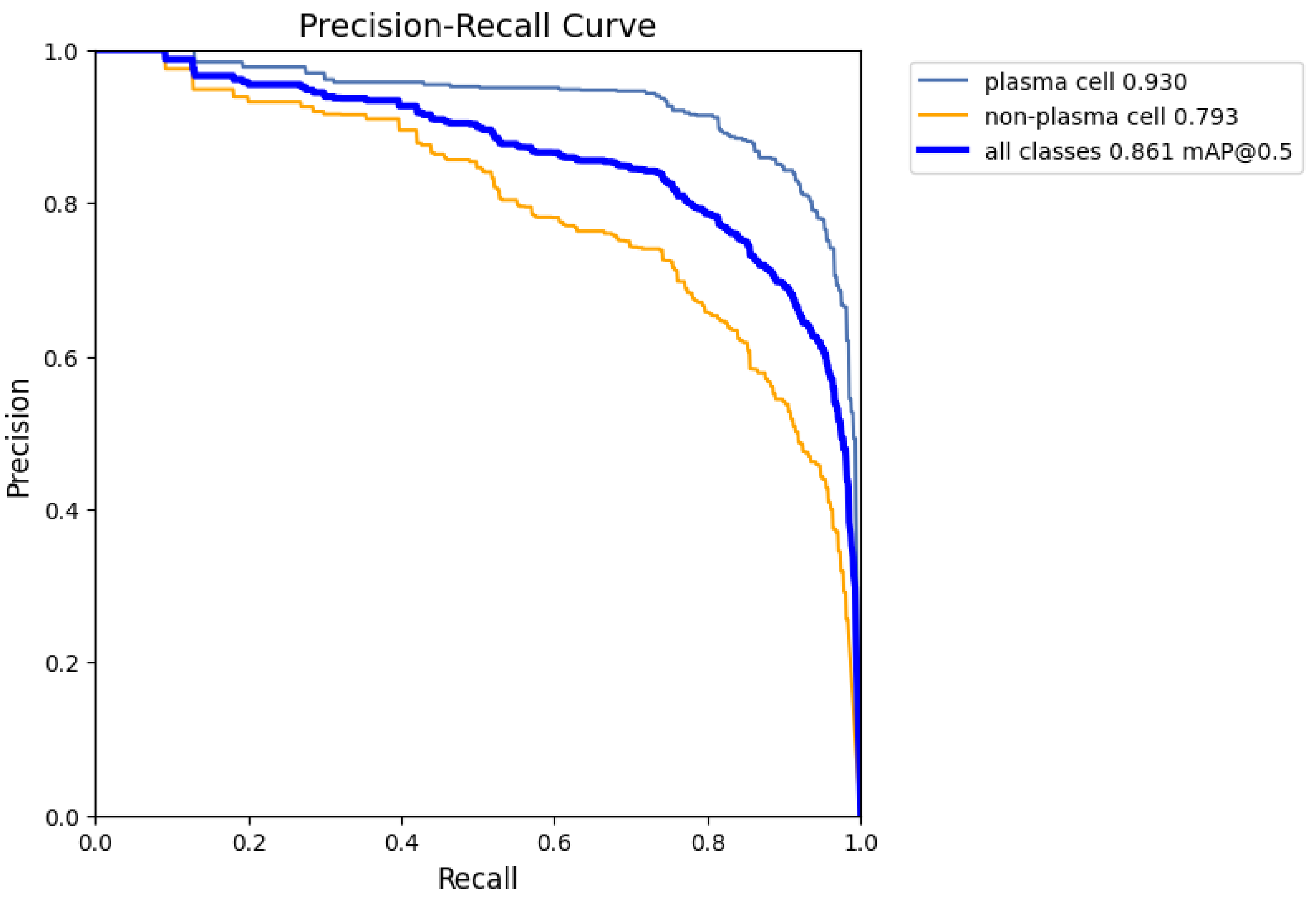

Figure 6. Precision and recall curve to illustrate the learning process.

2.2 Fold 2

2.2.1 Label and bounding box distributions

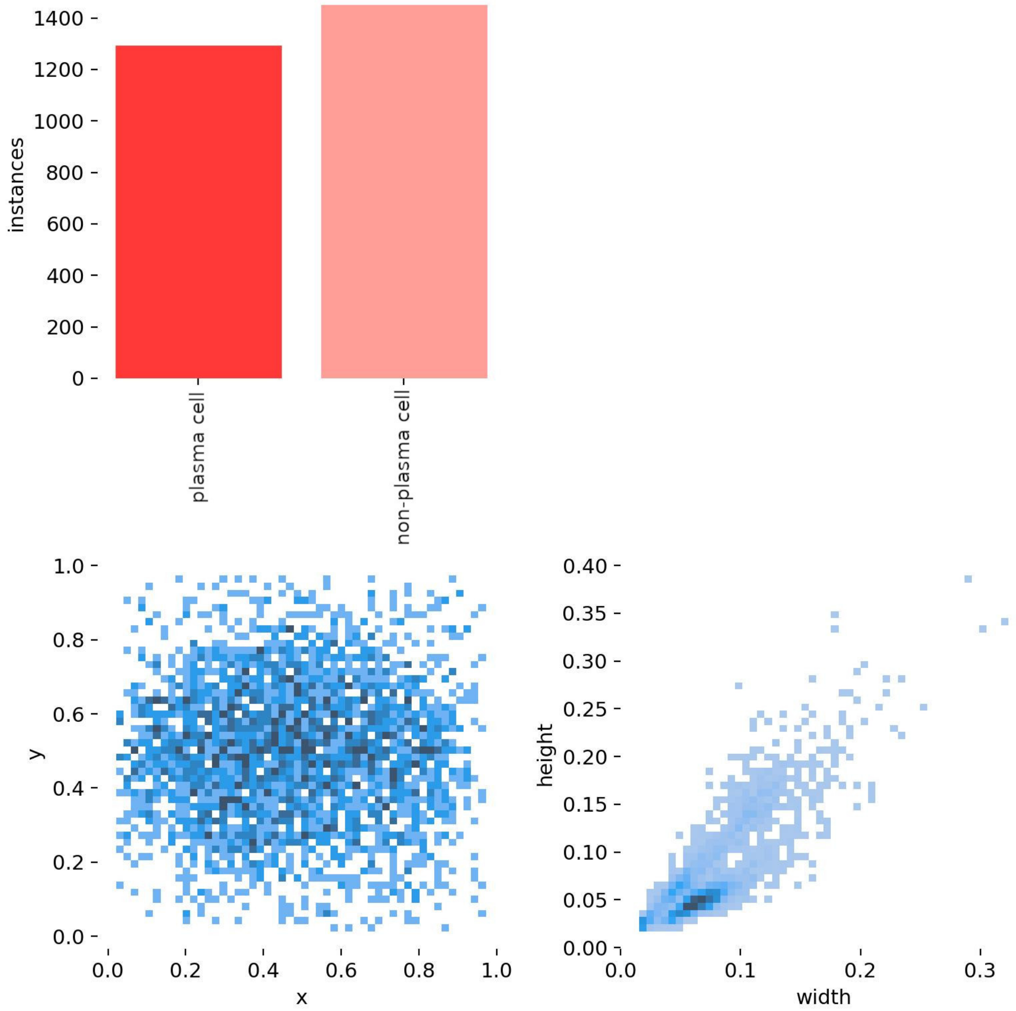

Figure 7. Label and bounding box distributions over slide images.

2.2.2 F1-score Results

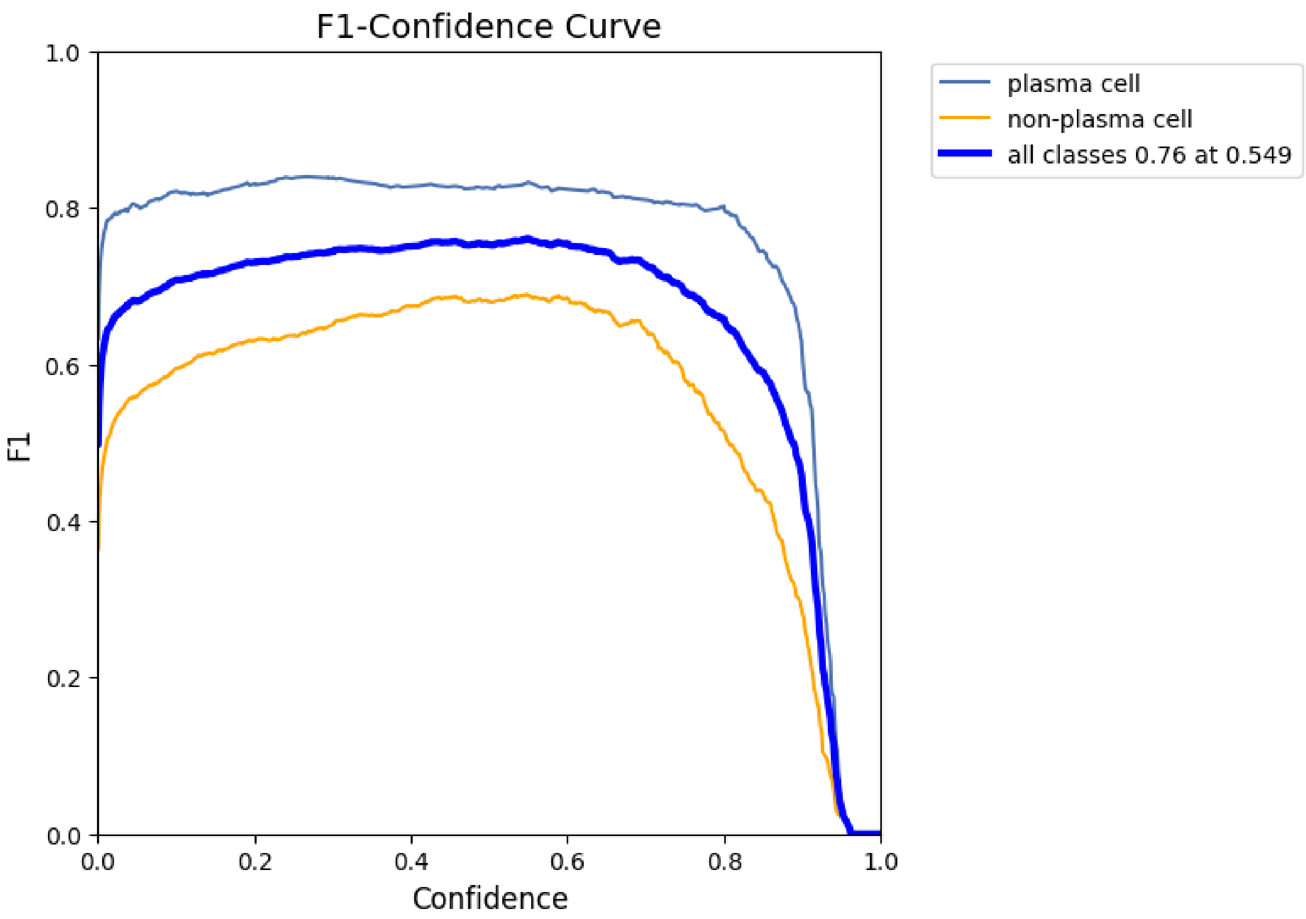

Figure 8. Best F1-score obtained by varying the confidence threshold.

2.2.3 Confusion Matrix Results

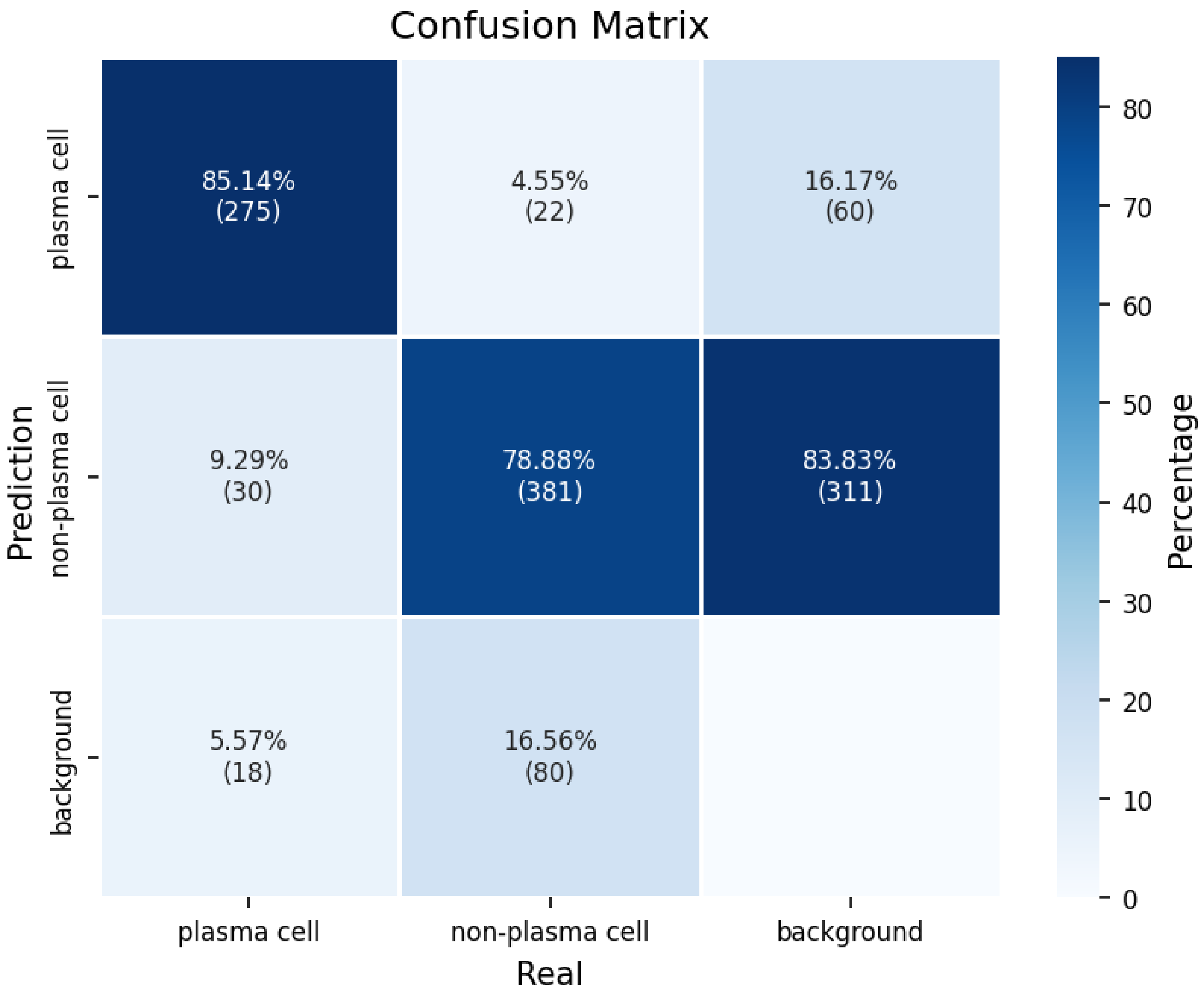

Figure 9. Confusion matrix summarizing the performance of our DNN model in each class.

2.2.4 Training Performance

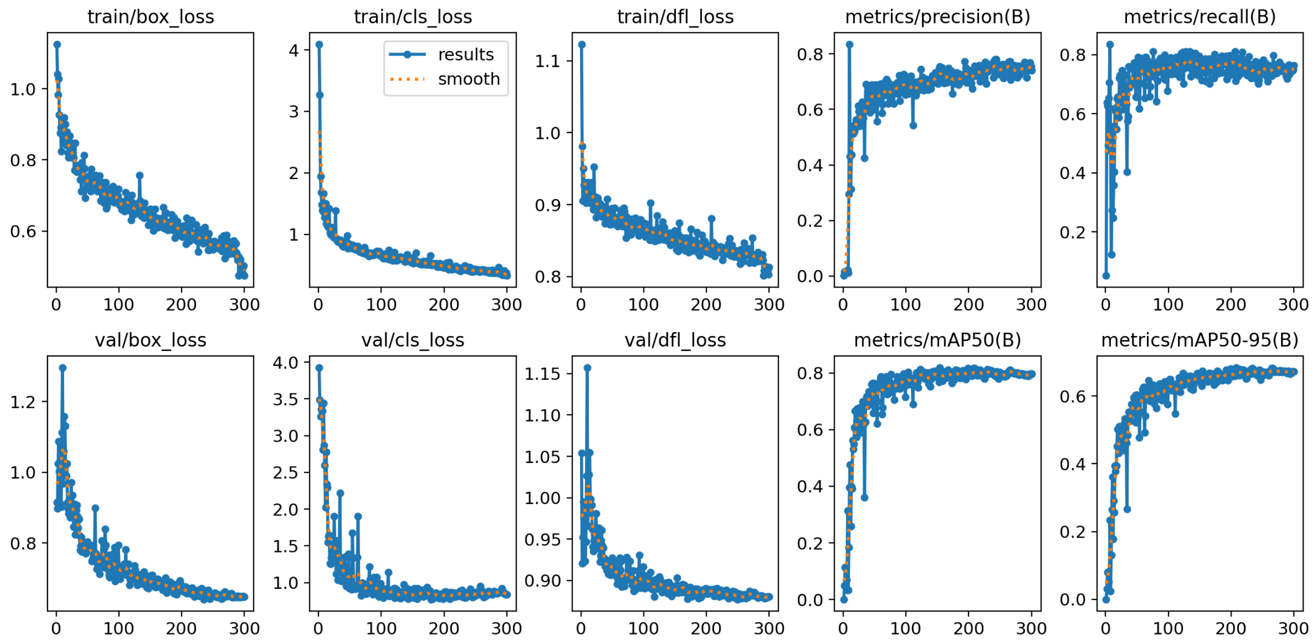

Figure 10. General training and validation performances.

2.2.5 Precision-Recall Curve

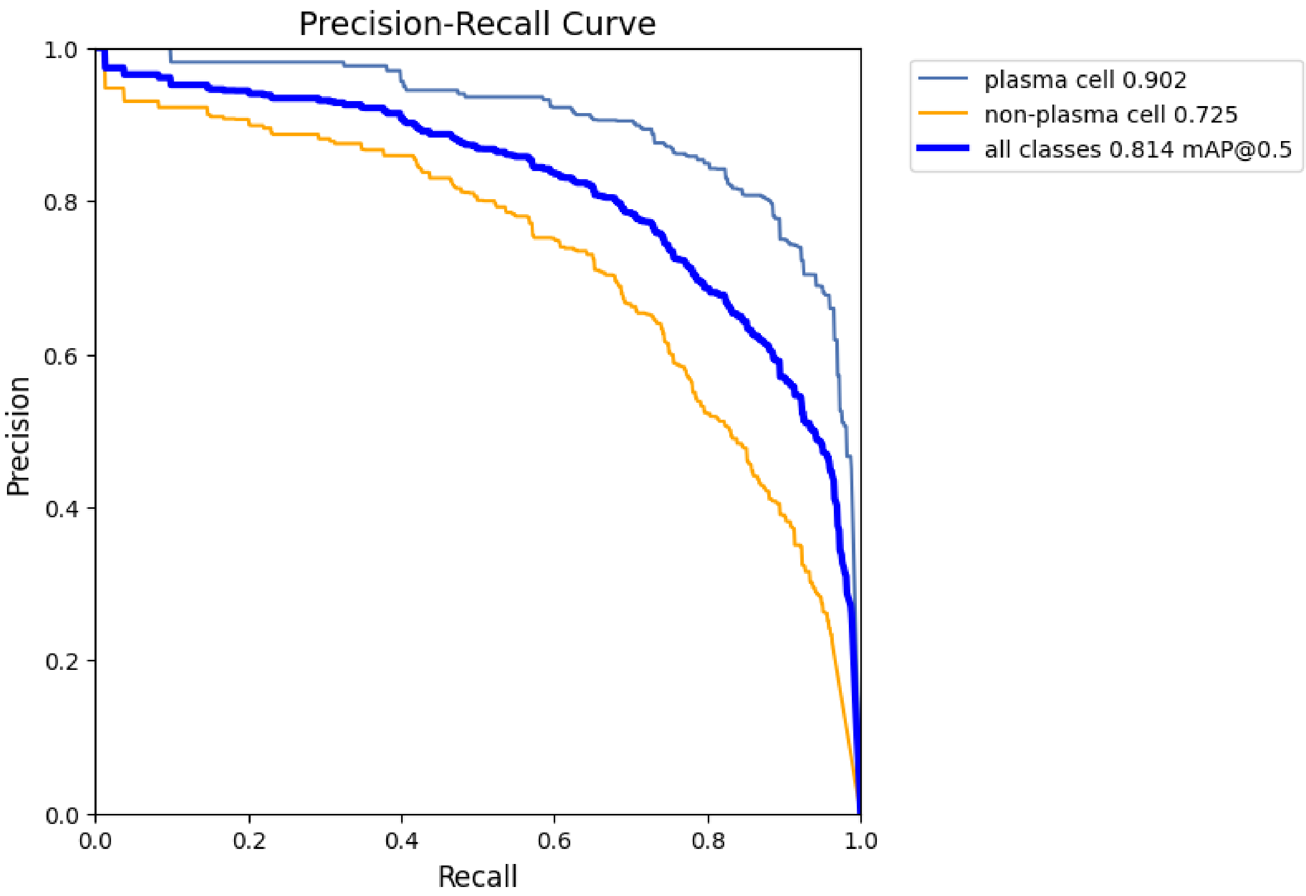

Figure 11. Precision and recall curve to illustrate the learning process.

2.3 Fold 3

2.3.1 Label and bounding box distributions

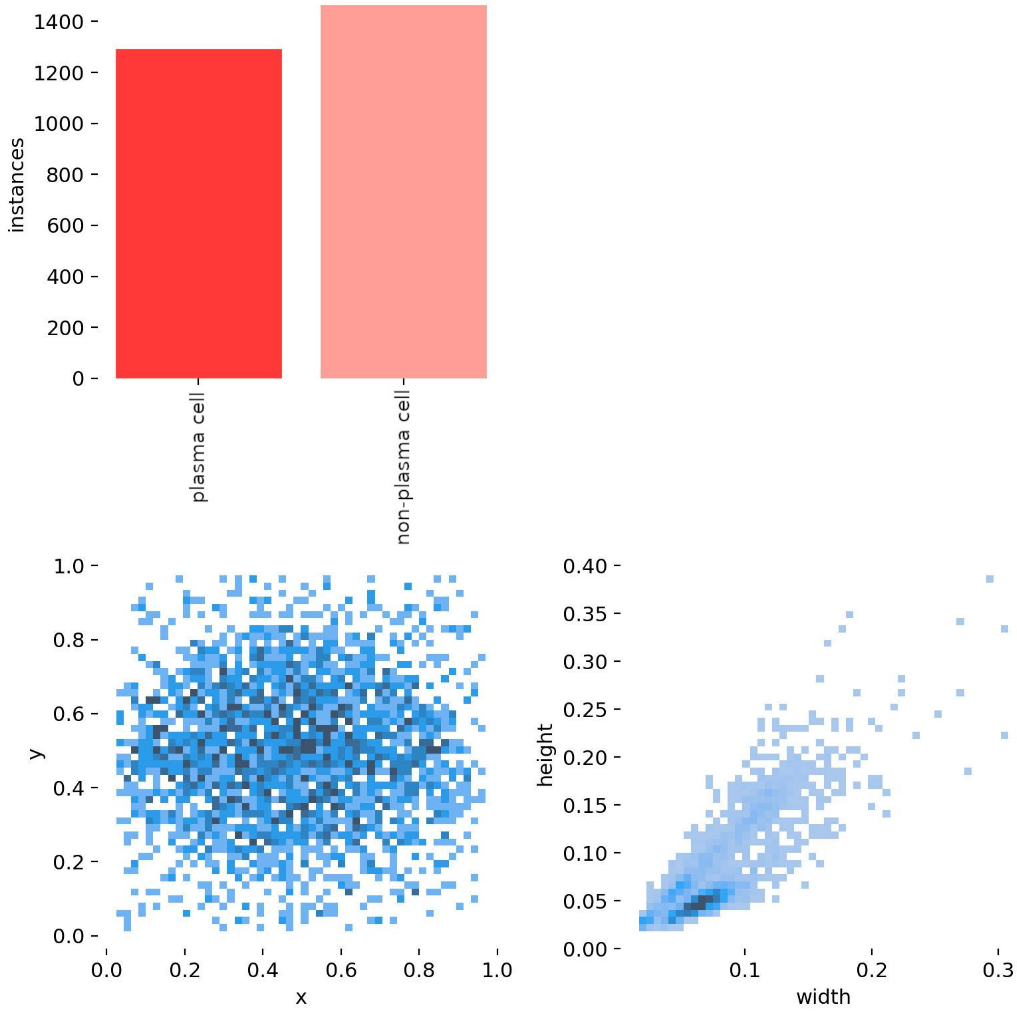

Figure 12. Label and bounding box distributions over slide images.

2.3.2 F1-score Results

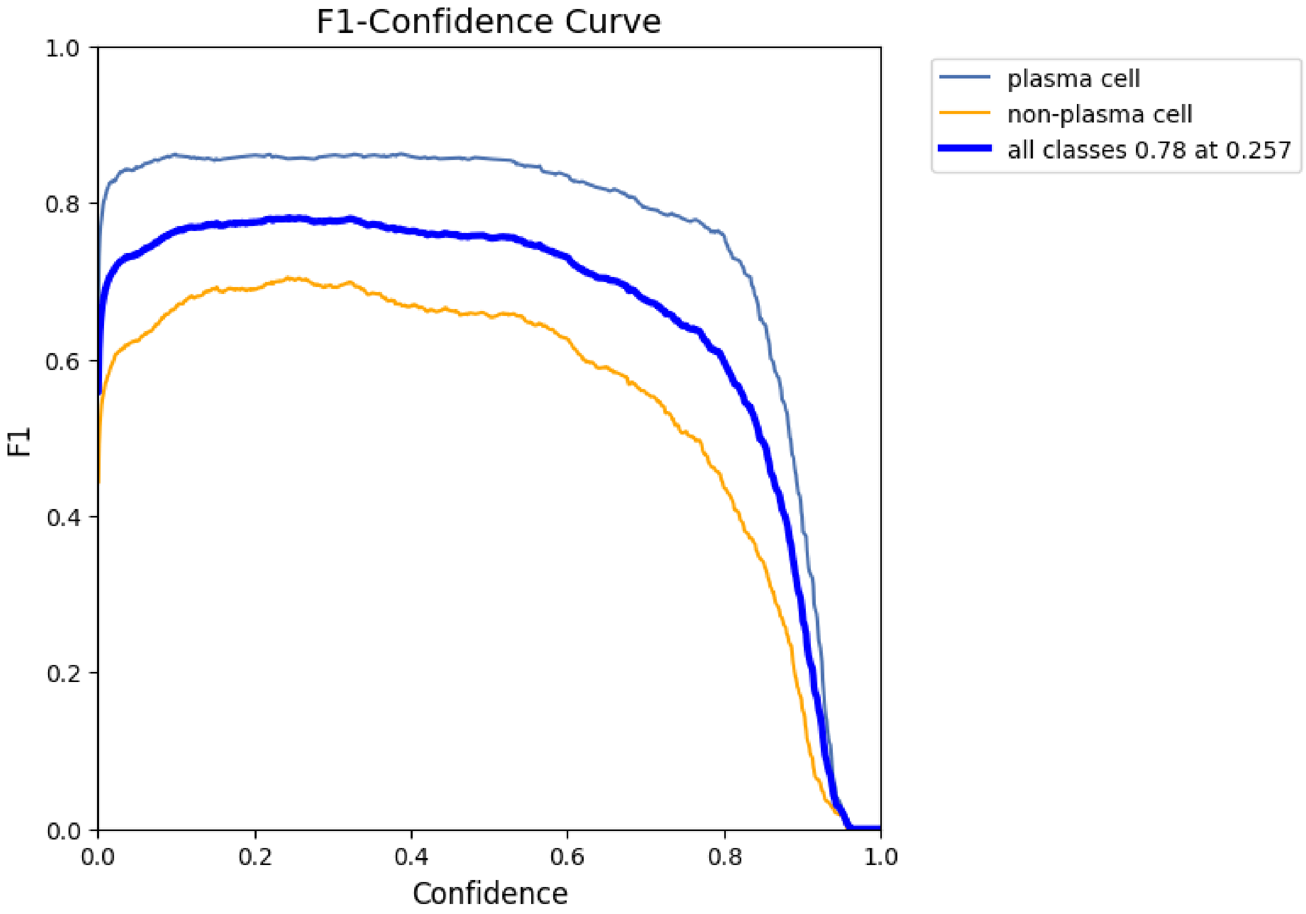

Figure 13. Best F1-score obtained by varying the confidence threshold.

2.3.3 Confusion Matrix Results

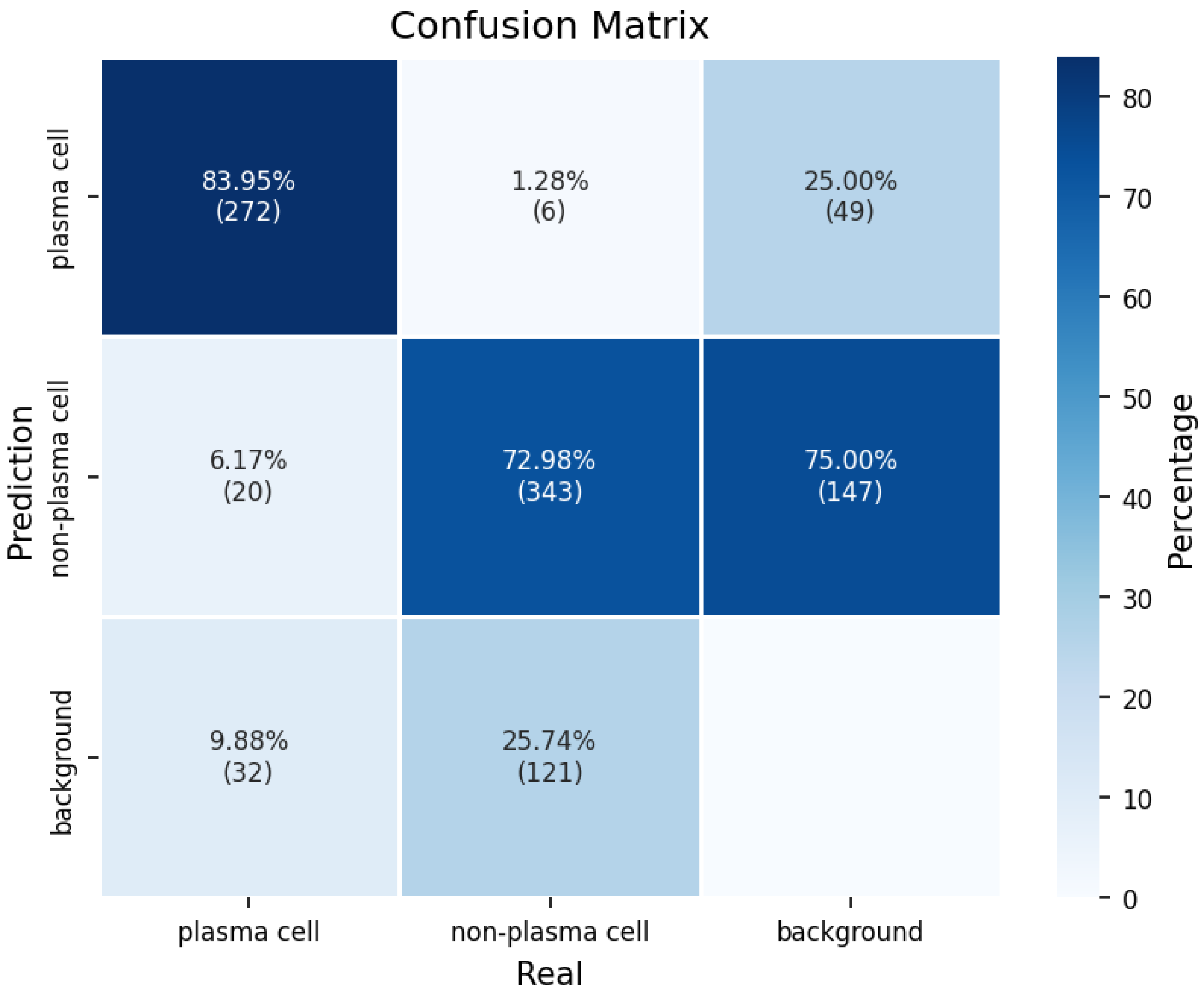

Figure 14. Confusion matrix summarizing the performance of our DNN model in each class.

2.3.4 Training Performance

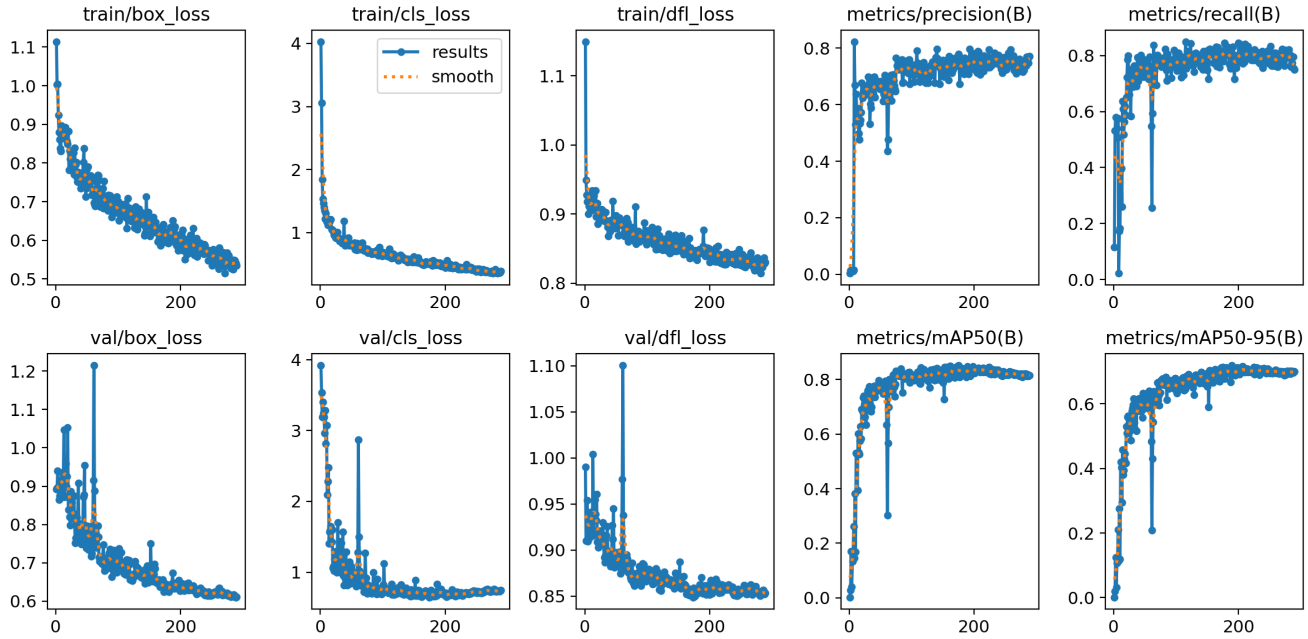

Figure 15. General training and validation performances.

2.3.5 Precision-Recall Curve

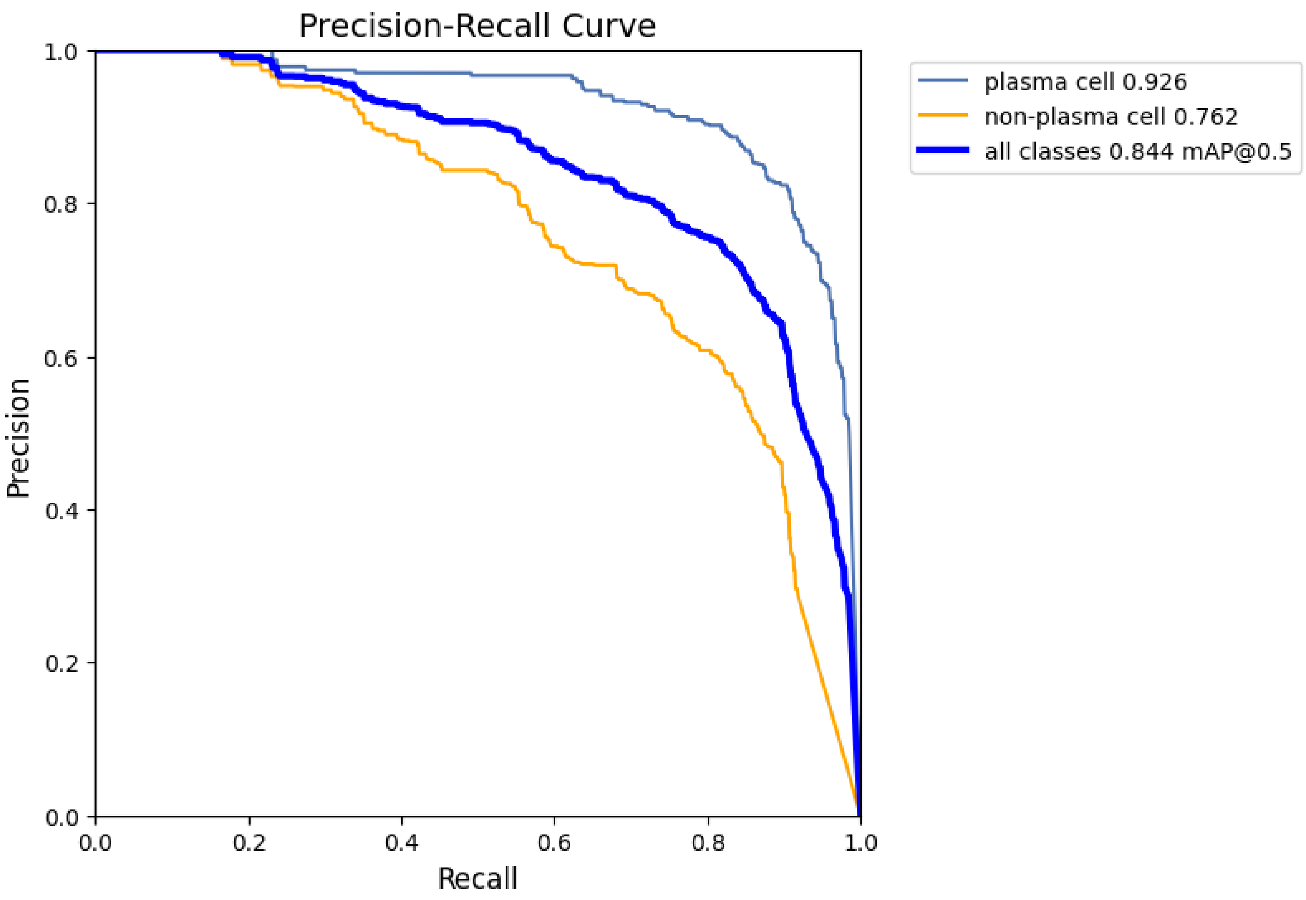

Figure 16. Precision and recall curve to illustrate the learning process.

2.4 Fold 4

2.4.1 Label and bounding box distributions

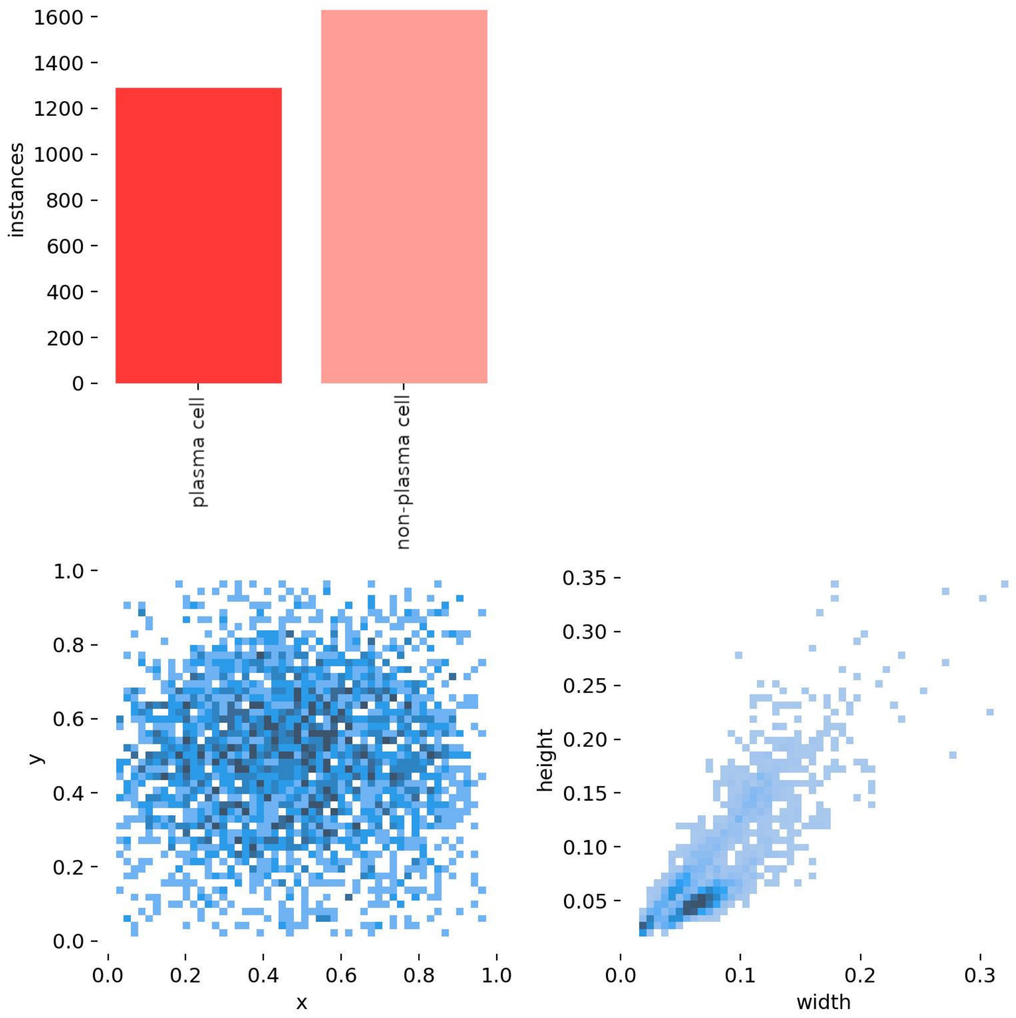

Figure 17. Label and bounding box distributions over slide images.

2.4.2 F1-score Results

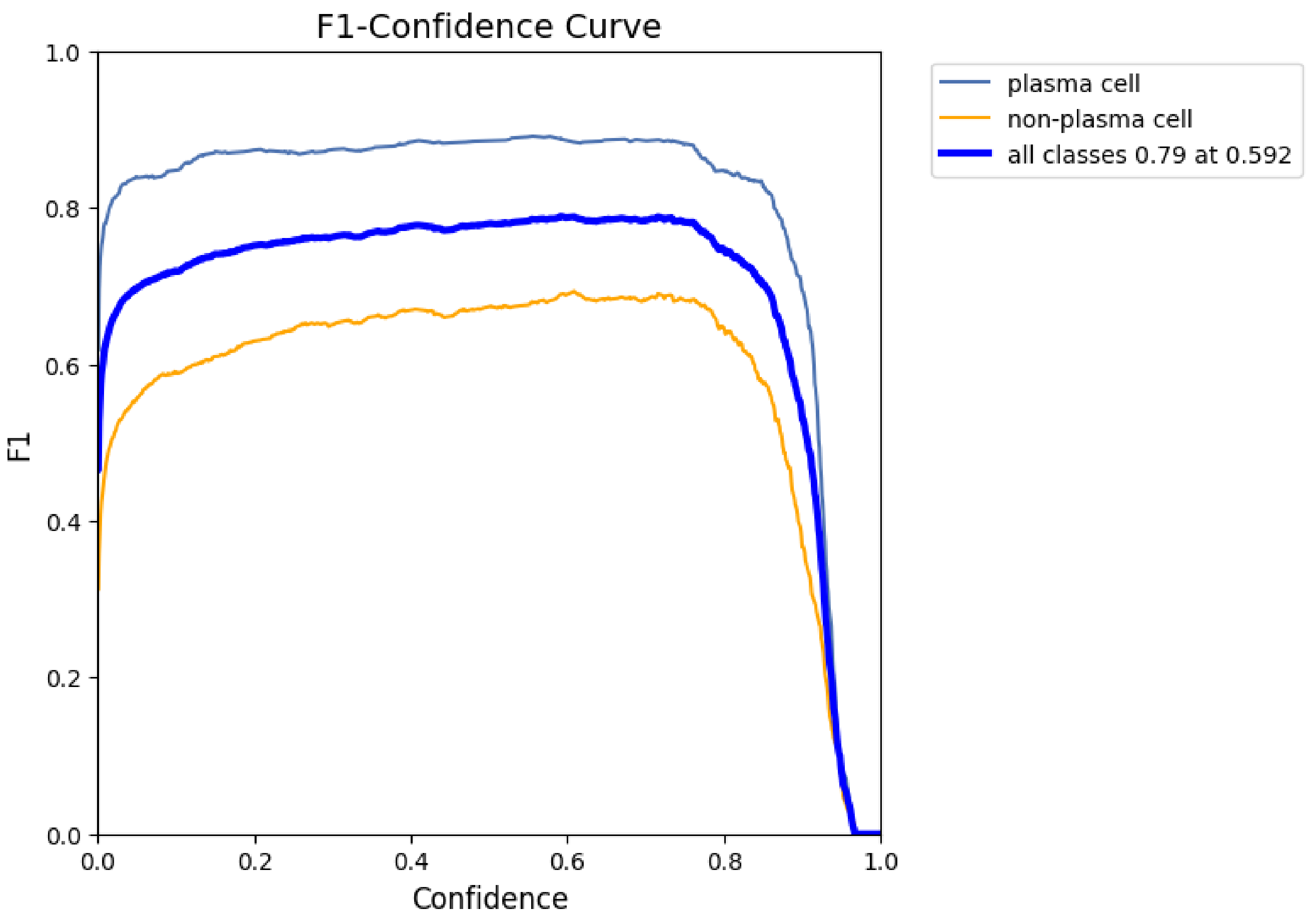

Figure 18. Best F1-score obtained by varying the confidence threshold.

2.4.3 Confusion Matrix Results

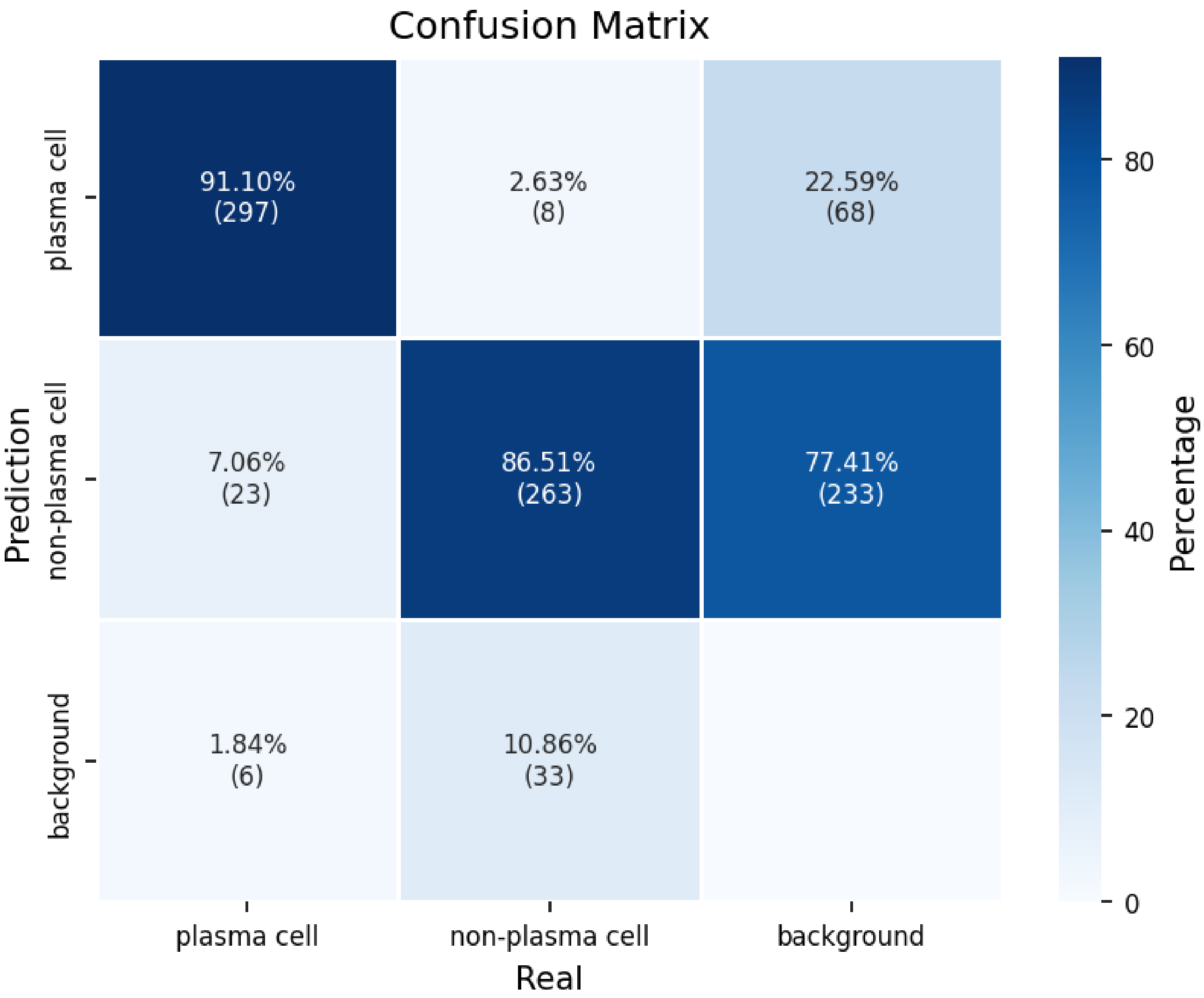

Figure 19. Confusion matrix summarizing the performance of our DNN model in each class.

2.4.4 Training Performance

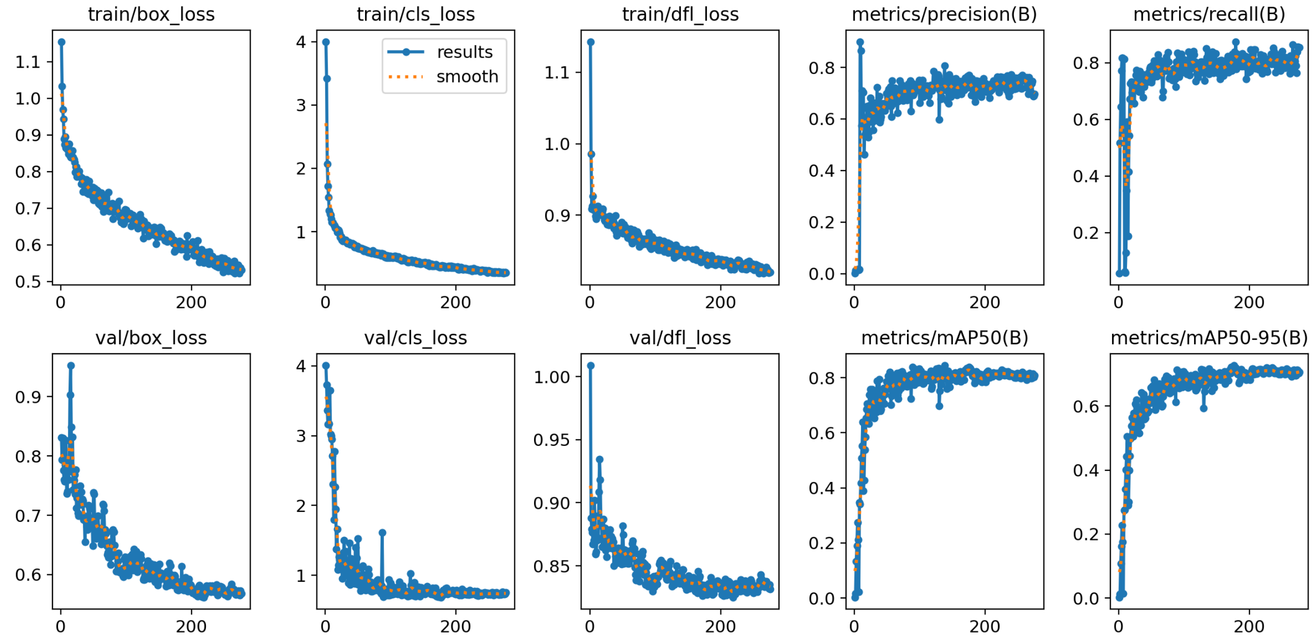

Figure 20. General training and validation performances.

2.4.5 Precision-Recall Curve

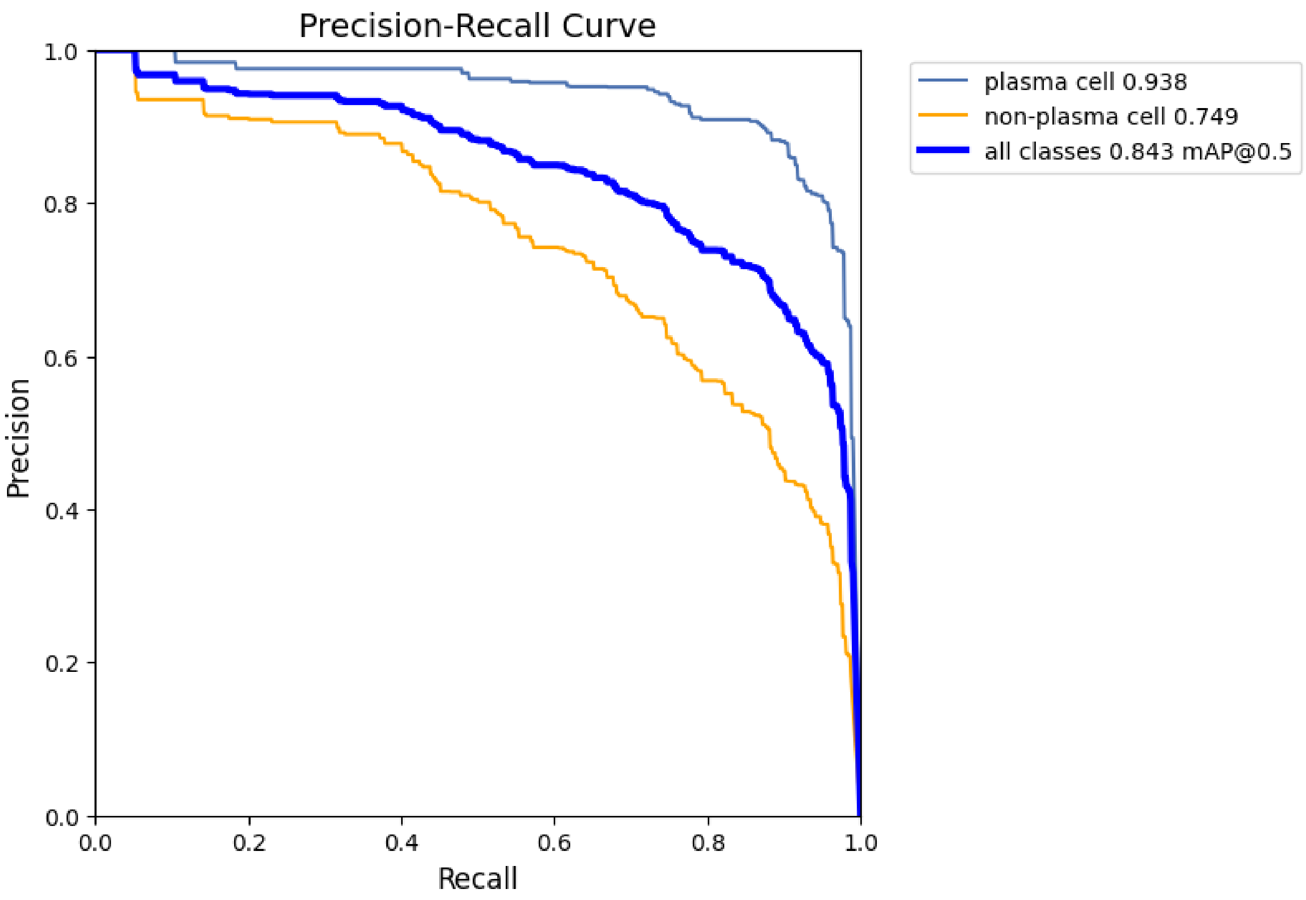

Figure 21. Precision and recall curve to illustrate the learning process.

2.5 Fold 5

2.5.1 Label and bounding box distributions

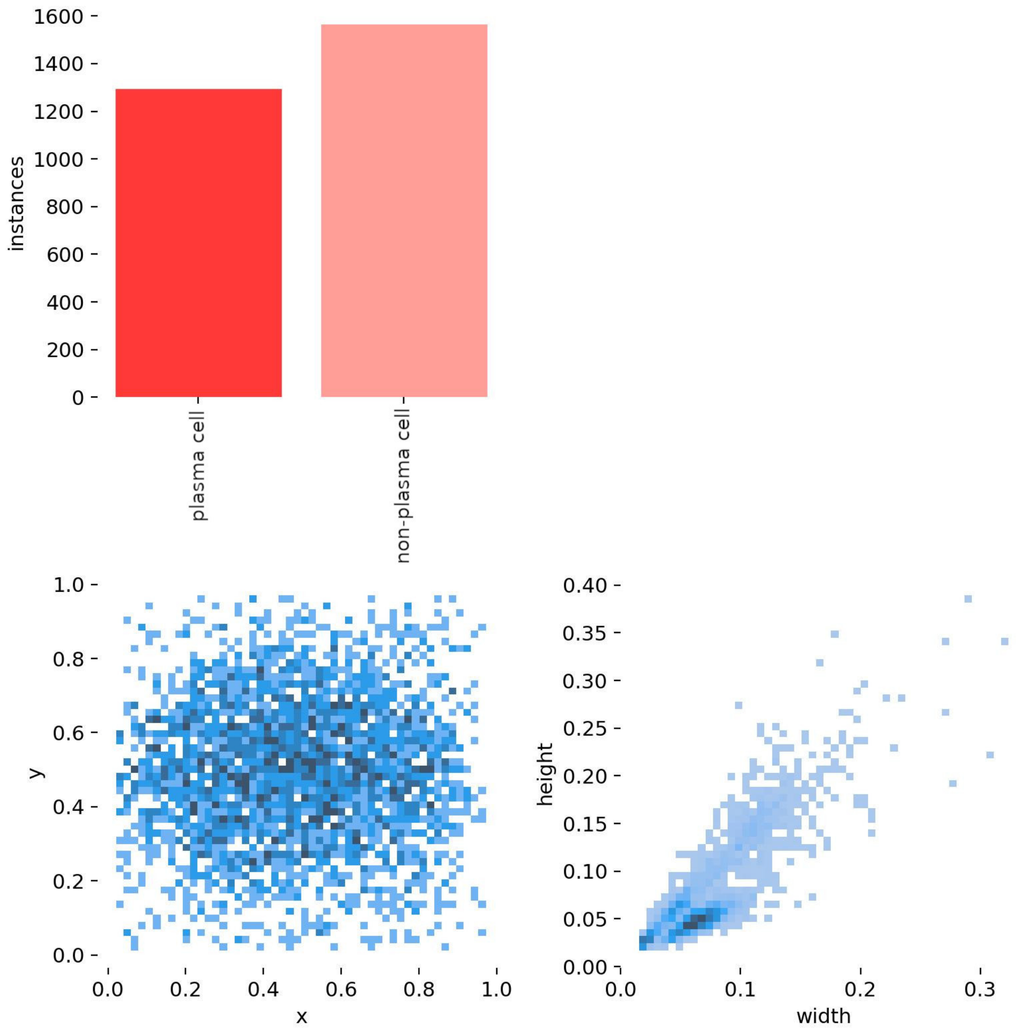

Figure 22. Label and bounding box distributions over slide images.

2.5.2 F1-score Results

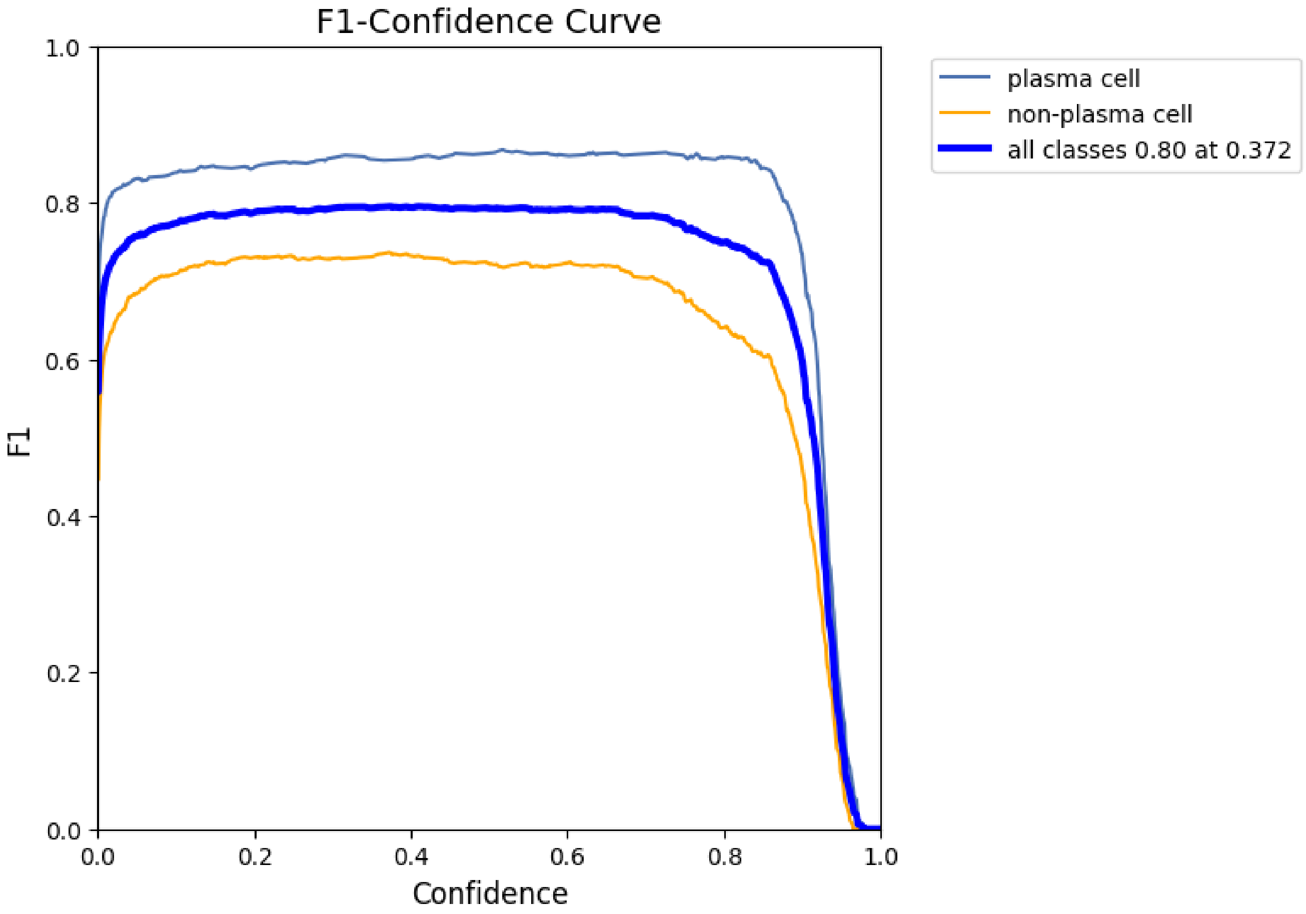

Figure 23. Best F1-score obtained by varying the confidence threshold.

2.5.3 Confusion Matrix Results

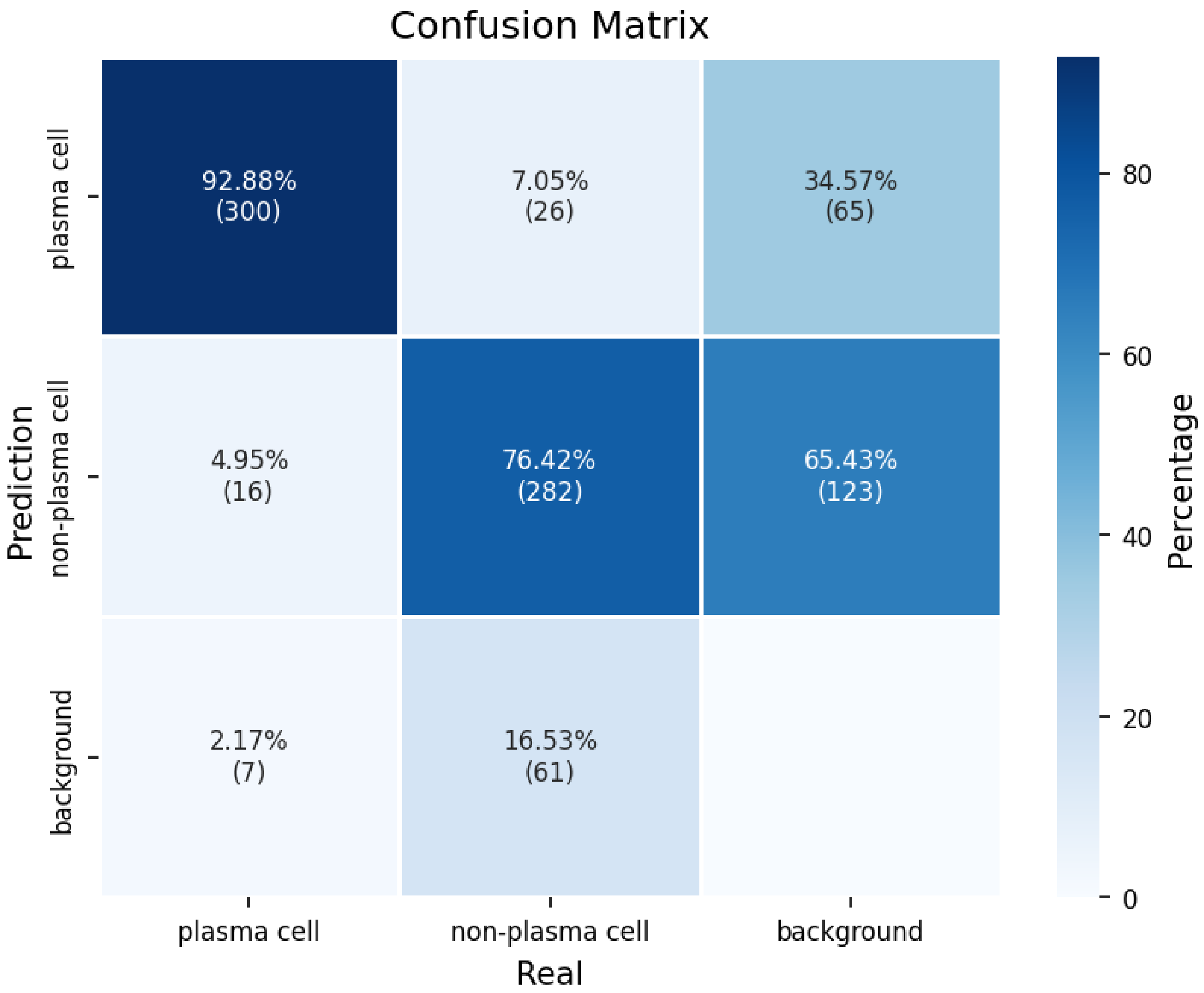

Figure 24. Confusion matrix summarizing the performance of our DNN model in each class.

2.5.4 Training Performance

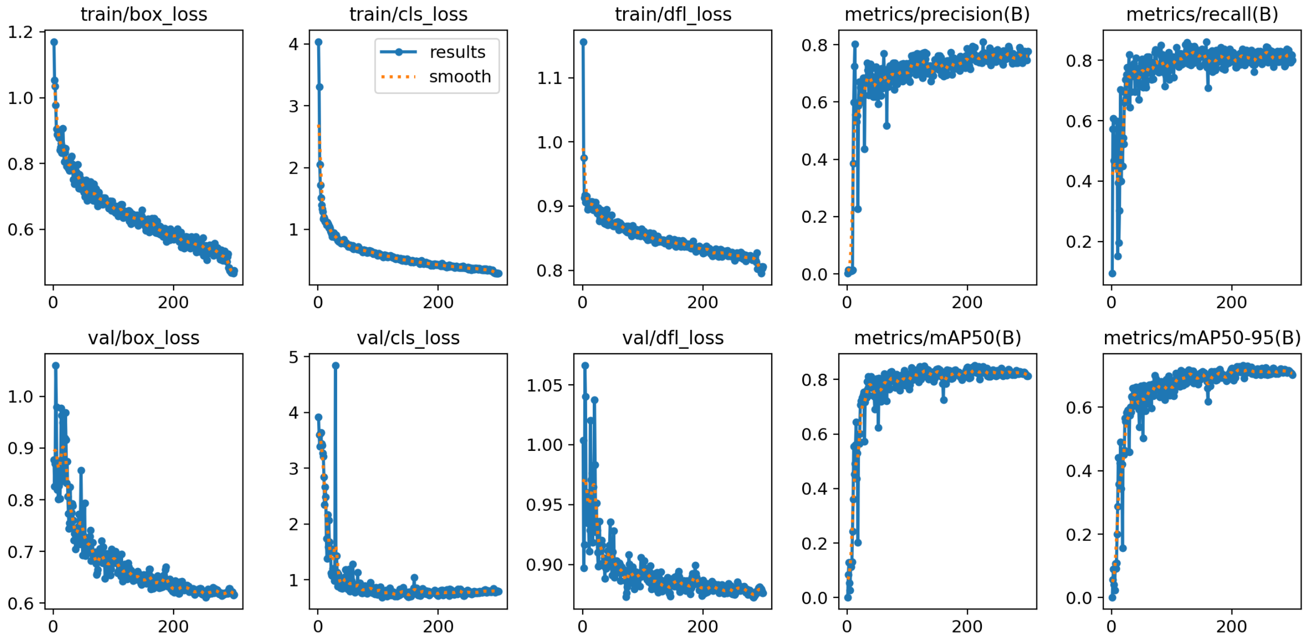

Figure 25. General training and validation performances.

2.5.5 Precision-Recall Curve

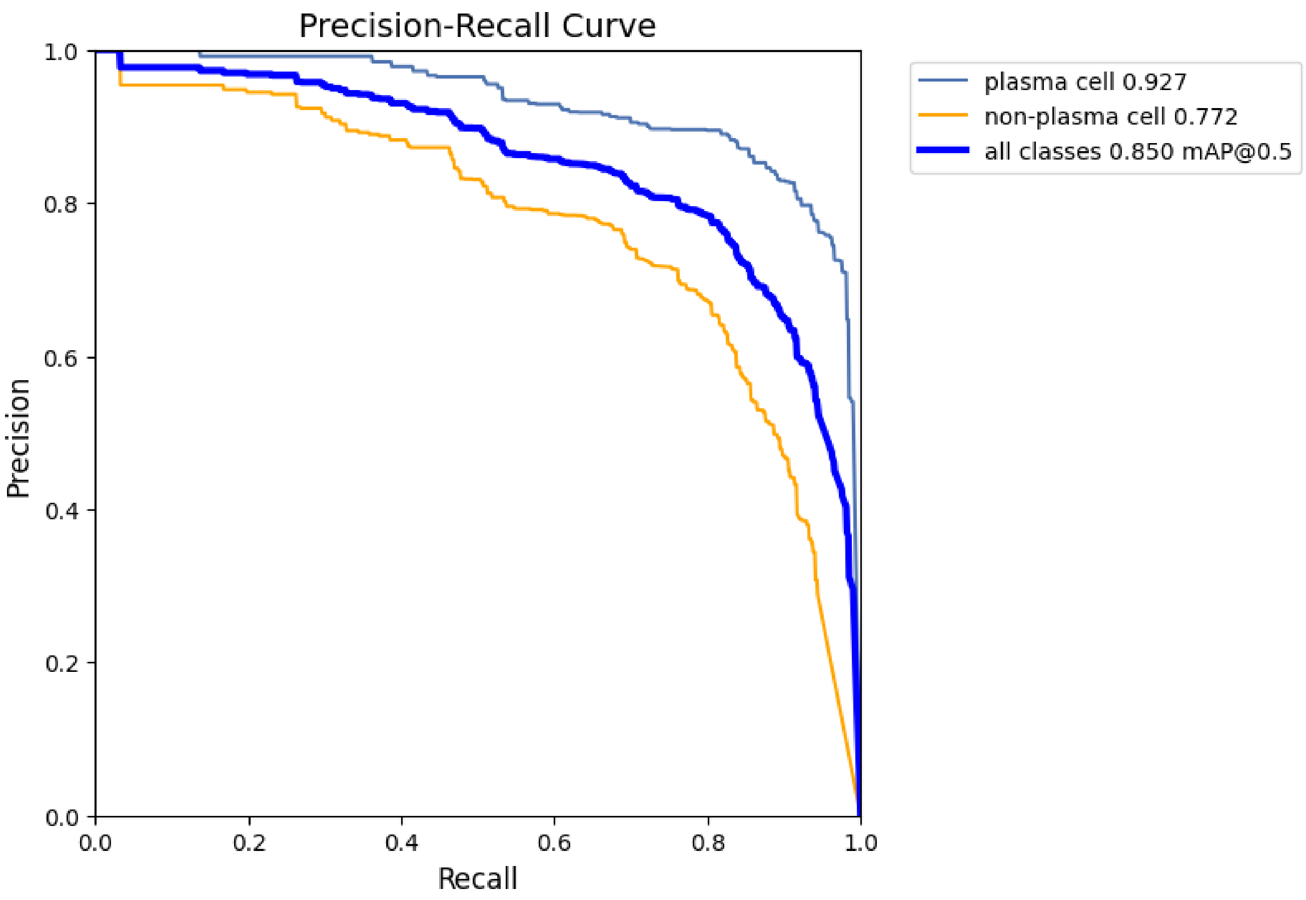

Figure 26. Precision and recall curve to illustrate the learning process.
